# Supplementary material for: Holobiome Structure and Microbial Core Assemblages of Deschampsia antarctica Across the South Shetland Islands
Source: Plants (Basel). 2025 Nov 30;14(23):3657. doi: 10.3390/plants14233657 (PMC12693956; doi:10.3390/plants14233657)
Supplement: Supplementary file 1 [file plants-14-03657-s001.zip › plants-4002576-supplementary.pdf]

Supplementary information

Supplementary Table S1. Summary of paired-end sequencing read processing, showing initial raw read pairs, merged reads with percentage, reads passing expected errors (EE ≤ 1) in quality control (QC_ee), final usable reads after quality control, and percentage retained, for each sample.

SampleID	*Raw reads*	*merged*	*(%)*	*QC_ee*	*final*	*(%)*
Ar1BS	108866	82360	75.65	78783	76887	**70.63**
Ar1PA	1256174	1028825	81.90	1009645	697120	**55.50**
Ar1RH	1255538	1098648	87.50	1078377	821394	**65.42**
Ar2BS	86033	59171	68.78	56698	55530	**64.55**
Ar2PA	1379995	1119699	81.14	1096995	753740	**54.62**
Ar2RH	1490332	1312060	88.04	1285177	980512	**65.79**
Ar3BS	108563	84265	77.62	80662	78636	**72.43**
Ar3PA	1449717	1195780	82.48	1170422	804269	**55.48**
Ar3RH	1072879	928786	86.57	910560	711439	**66.31**
Ar4BS	103236	78208	75.76	74916	73041	**70.75**
Ar4PA	1317375	1069459	81.18	1046422	715386	**54.30**
Ar4RH	1274886	1053732	82.65	1029782	744892	**58.43**
Co1BS	103277	72513	70.21	69207	67644	**65.50**
Co1PA	1432208	1162345	81.16	1135881	787966	**55.02**
Co1RH	1013514	716753	70.72	698672	414022	**40.85**
Co2BS	129337	100743	77.89	96046	93937	**72.63**
Co2PA	1209124	969249	80.16	948530	653835	**54.08**
Co2RH	995700	793211	79.66	775786	496125	**49.83**
Co3BS	83343	60375	72.44	57778	56556	**67.86**
Co3PA	1221330	946325	77.48	926672	606146	**49.63**
Co3RH	626457	429273	68.52	420096	211441	**33.75**
Co4BS	86359	65483	75.83	62572	61217	**70.89**
Co4PA	1551402	1268270	81.75	1243095	884700	**57.03**
Co4RH	223461	138256	61.87	135210	48017	**21.49**
Li1BS	89014	59907	67.30	57096	55663	**62.53**
Li1PA	1394720	1206955	86.54	1184090	882240	**63.26**
Li1RH	1103697	976148	88.44	957724	751792	**68.12**
Li2PA	1188103	967490	81.43	947926	664816	**55.96**
Li2RH	942524	832288	88.30	815652	629085	**66.74**
Li3BS	105302	78490	74.54	74707	72980	**69.31**
Li3PA	1172014	981297	83.73	961558	673638	**57.48**
Li3RH	1005976	869489	86.43	848414	664288	**66.03**
Li4BS	93275	65479	70.20	62313	60945	**65.34**
Li4PA	1174424	937190	79.80	916735	618215	**52.64**
Li4RH	1017935	891657	87.59	870089	680494	**66.85**

Supplementary Table S2. Summary of paired-end sequencing read processing, showing initial raw read pairs, ITSexpress reads with percentage, merged reads and reads passing expected errors (EE ≤ 1) in quality control (QC_ee), and percentage retained, for each sample.

SampleID	*Raw reads*	*ITSexpress*	*(%)*	*Merged*	*QC_ee*	*(%)*
Ar1BS	176717	170407	96.43	170251	169367	**95.84**
Ar1RH	125598	122122	97.23	121865	37989	**30.25**
Ar2BS	206781	198376	95.94	198240	197270	**95.40**
Ar2PA	210122	189254	90.07	188599	173132	**82.40**
Ar2RH	141658	132767	93.72	132361	126539	**89.33**
Ar3BS	204007	195443	95.80	195269	194041	**95.11**
Ar3PA	219580	205612	93.64	205217	198271	**90.30**
Ar3RH	181268	164858	90.95	164097	145630	**80.34**
Ar4BS	204388	196454	96.12	196281	195200	**95.50**
Ar4PA	205725	190069	92.39	189468	181450	**88.20**
Ar4RH	151520	137342	90.64	136856	125365	**82.74**
Co1BS	202846	191415	94.36	191110	190083	**93.71**
Co1PA	216412	205890	95.14	205622	201868	**93.28**
Co1RH	198676	183922	92.57	183554	179368	**90.28**
Co2BS	202144	188124	93.06	187771	186718	**92.37**
Co2PA	214669	202874	94.51	202574	199265	**92.82**
Co2RH	210669	160075	75.98	159876	157630	**74.82**
Co3BS	196056	180452	92.04	179782	178374	**90.98**
Co3PA	186250	172143	92.43	171778	166038	**89.15**
Co3RH	203894	195567	95.92	195348	192711	**94.52**
Co4BS	62671	58890	93.97	58472	57733	**92.12**
Co4PA	185167	167929	90.69	166061	157672	**85.15**
Co4RH	206564	191317	92.62	190596	184450	**89.29**
Li1BS	202962	190582	93.90	190184	188731	**92.99**
Li1PA	100449	94122	93.70	93868	90488	**90.08**
Li1RH	204240	184890	90.53	183440	181186	**88.71**
Li2BS	203994	195150	95.66	194834	193580	**94.89**
Li2PA	159544	152899	95.84	152728	149628	**93.78**
Li2RH	203297	191876	94.38	191587	186763	**91.87**
Li3BS	161286	154112	95.55	153797	145511	**90.22**
Li3PA	204391	195720	95.76	195373	191822	**93.85**
Li3RH	139613	130944	93.79	130151	127963	**91.66**
Li4BS	205266	194831	94.92	194345	192878	**93.96**
Li4PA	205507	188297	91.63	187350	180638	**87.90**
Li4RH	202284	193375	95.60	193100	189217	**93.54**

Supplementary Table S3. Network-level metrics of bacterial co-occurrence networks in *Deschampsia antarctica*. Values represent the number of nodes and edges, network density, mean degree, transitivity, and modularity for each compartment .(Rhizosphere, Root endosphere, and Leaf endosphere).

Compartment	Nodes	Edges	Density	Average Degree	Transitivity	Modularity
Rhizosphere	168	520	0.04	6.19	0.41	0.60
Root endosphere	188	752	0.04	8.00	0.45	0.58
Leaf endosphere	32	43	0.09	2.69	0.54	0.59

Supplementary Table S4. Permutation-based pairwise comparisons of node-level network metrics (degree, closeness, betweenness, and eigenvector centrality) among microbial co-occurrence networks from the Rhizosphere, Root endosphere, and Leaf endosphere of *Deschampsia antarctica*.

Metric	Comparison	Mean Difference	P-value
**Degree**	*Rhizosphere vs Root endosphere*	-1.8095	0.0062
	Rhizosphere vs Leaf endosphere	3.503	0.0004
	Root endosphere vs Leaf endosphere	5.3125	0.0001
**Closeness**	*Rhizosphere vs Root endosphere*	0.0207	0.3214
	Rhizosphere vs Leaf endosphere	-0.2261	0.0000
	Root endosphere vs Leaf endosphere	-0.2468	0.0000
**Betweenness**	*Rhizosphere vs Root endosphere*	0.0004	0.8579
	Rhizosphere vs Leaf endosphere	-0.0147	0.0324
	Root endosphere vs Leaf endosphere	-0.0151	0.0150
**Eigenvector**	*Rhizosphere vs Root endosphere*	-0.0247	0.3219
	Rhizosphere vs Leaf endosphere	-0.0837	0.0902
	Root endosphere vs Leaf endosphere	-0.059	0.2357

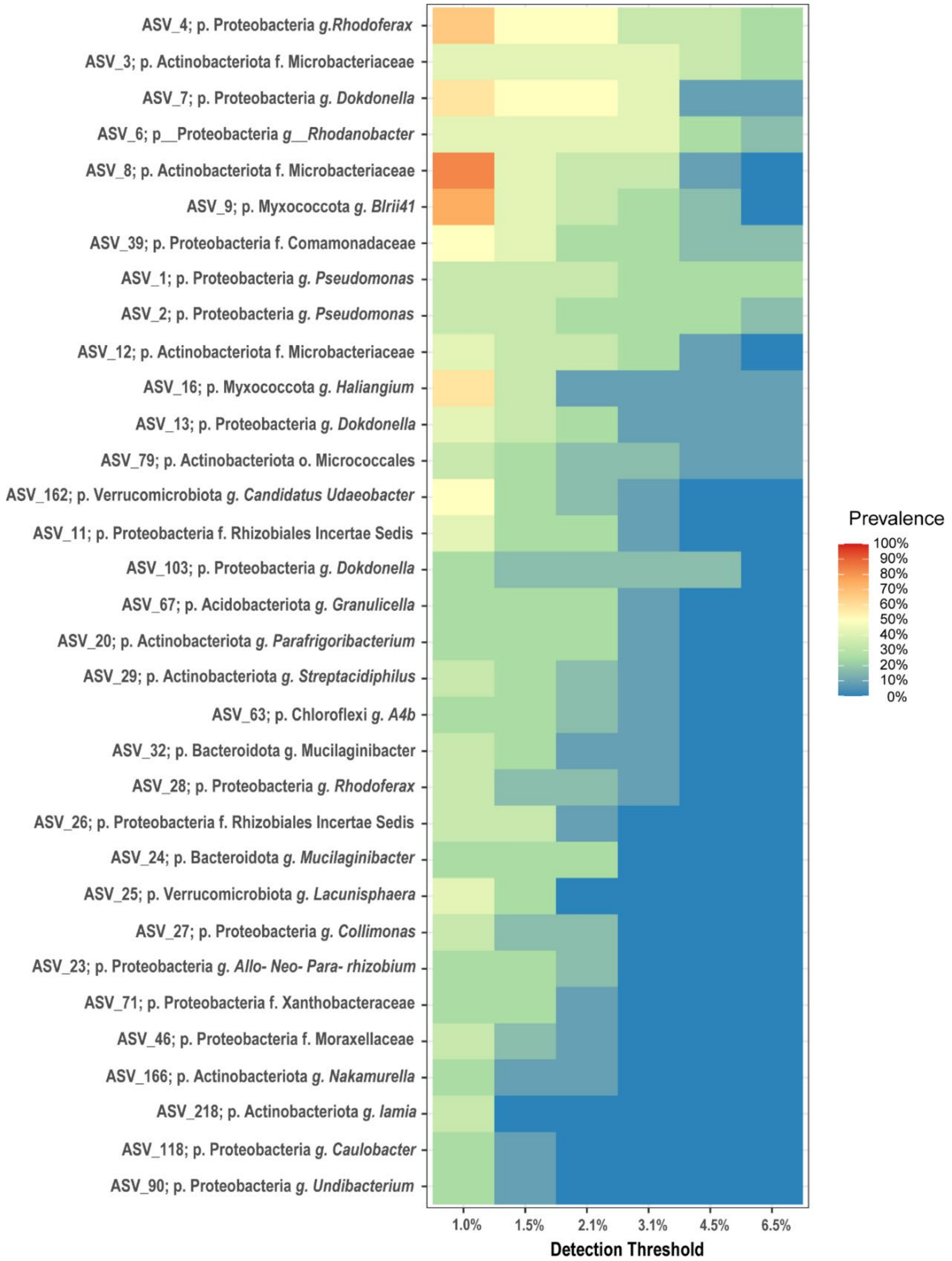

Supplementary Figure S1. Heatmap of zOTUS prevalence across detection thresholds, illustrating the relative distribution of bacterial genera according to their occurrence frequency in the root endosphere.

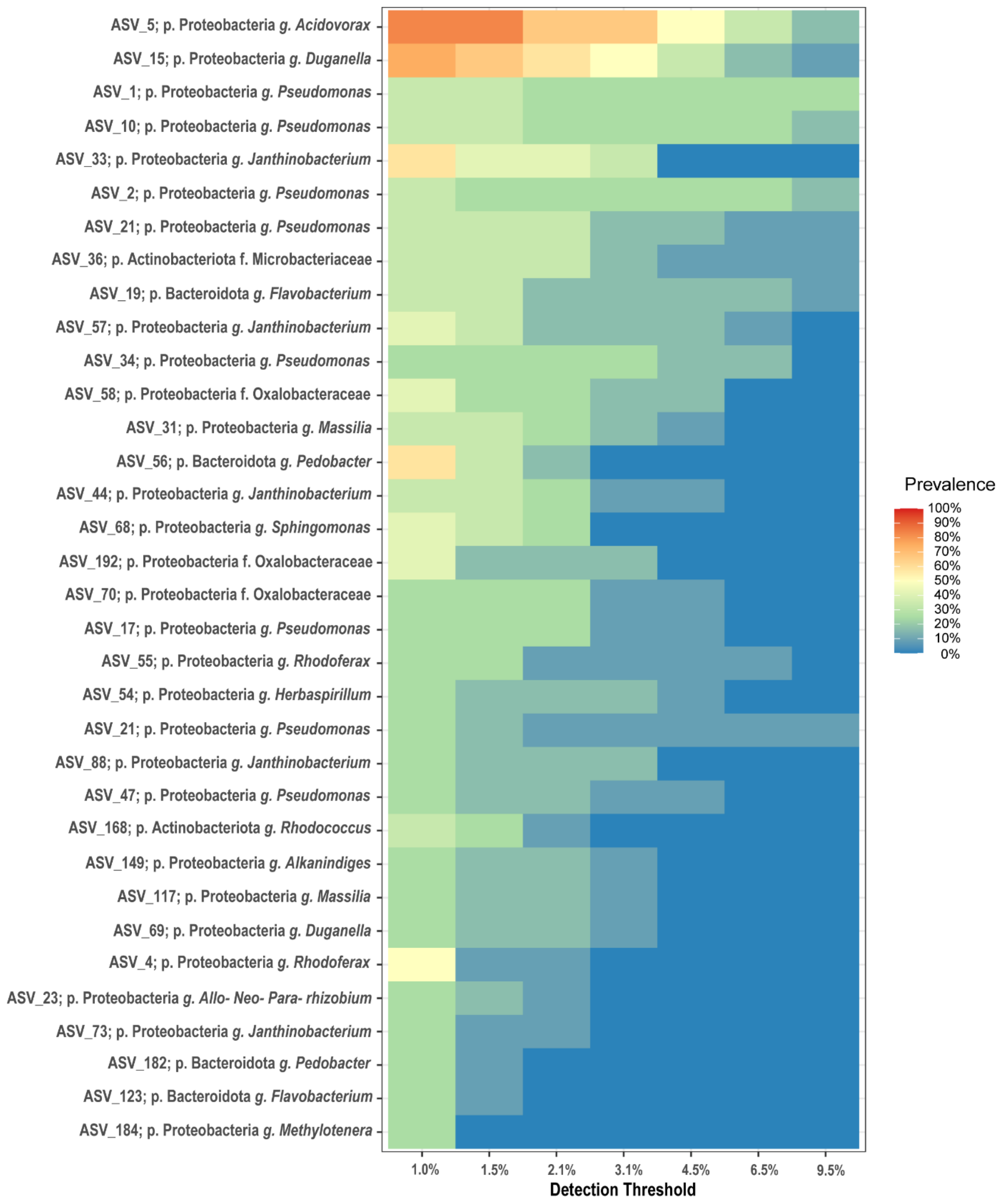

Supplementary Figure S2. Heatmap of zOTUS prevalence across detection thresholds, illustrating the relative distribution of bacterial genera according to their occurrence frequency in the leaf endosphere.

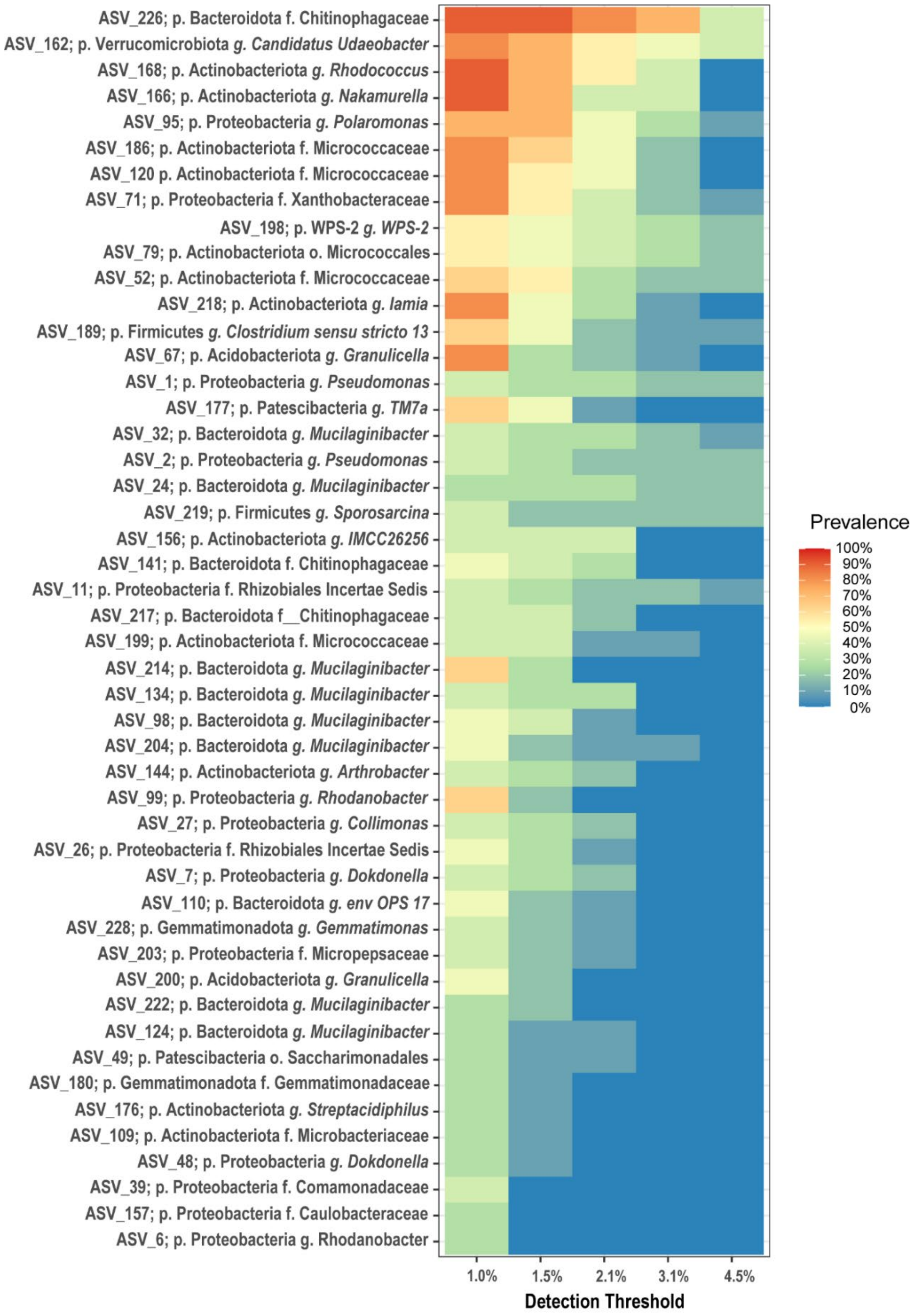

Supplementary Figure S3. Heatmap of zOTUS prevalence across detection thresholds, illustrating the relative distribution of bacterial genera according to their occurrence frequency in the rhizosphere.

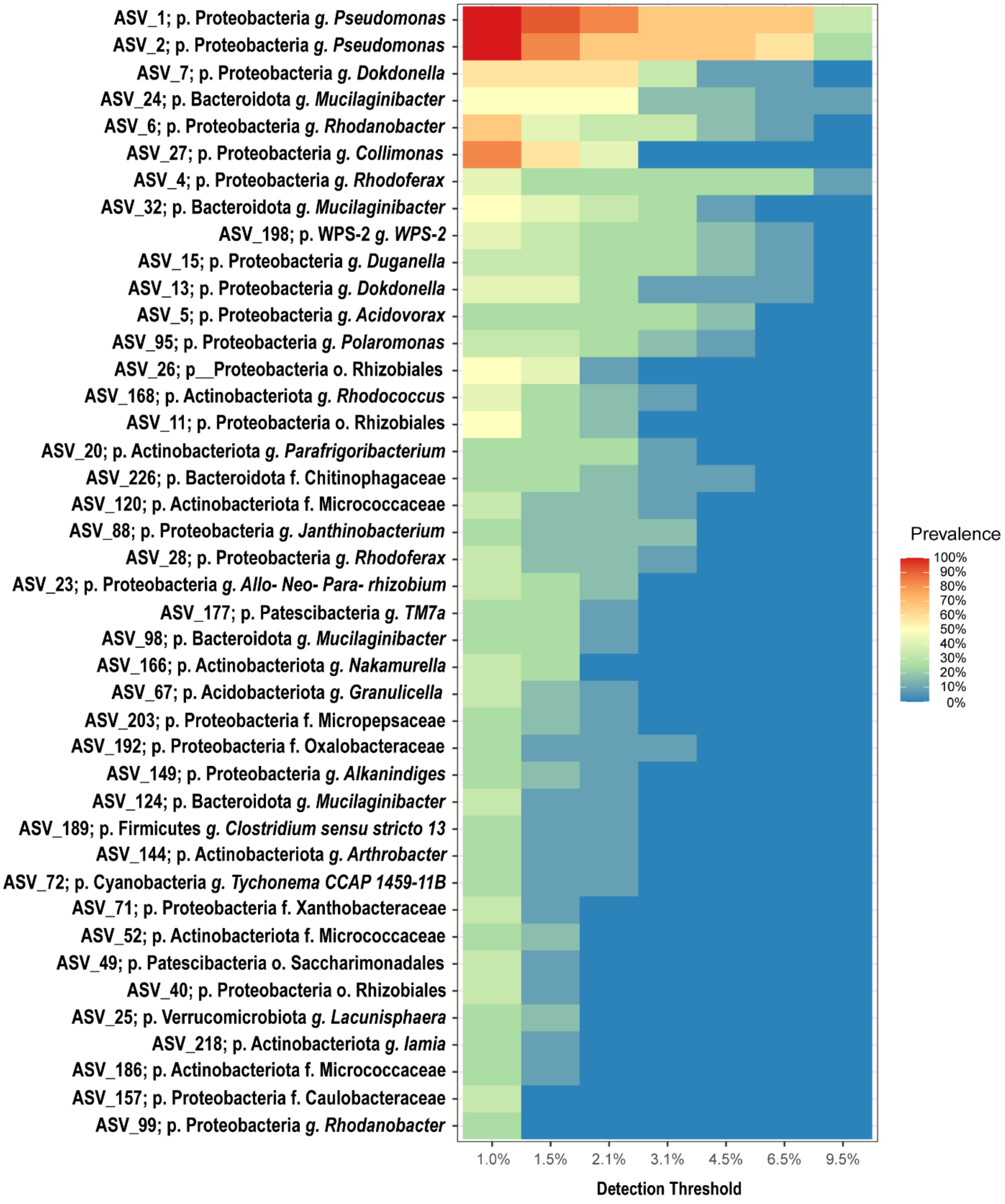

Supplementary Figure S4. Heatmap of zOTUS prevalence across detection thresholds, illustrating the relative distribution of bacterial genera according to their occurrence frequency in Admiralty Bay.

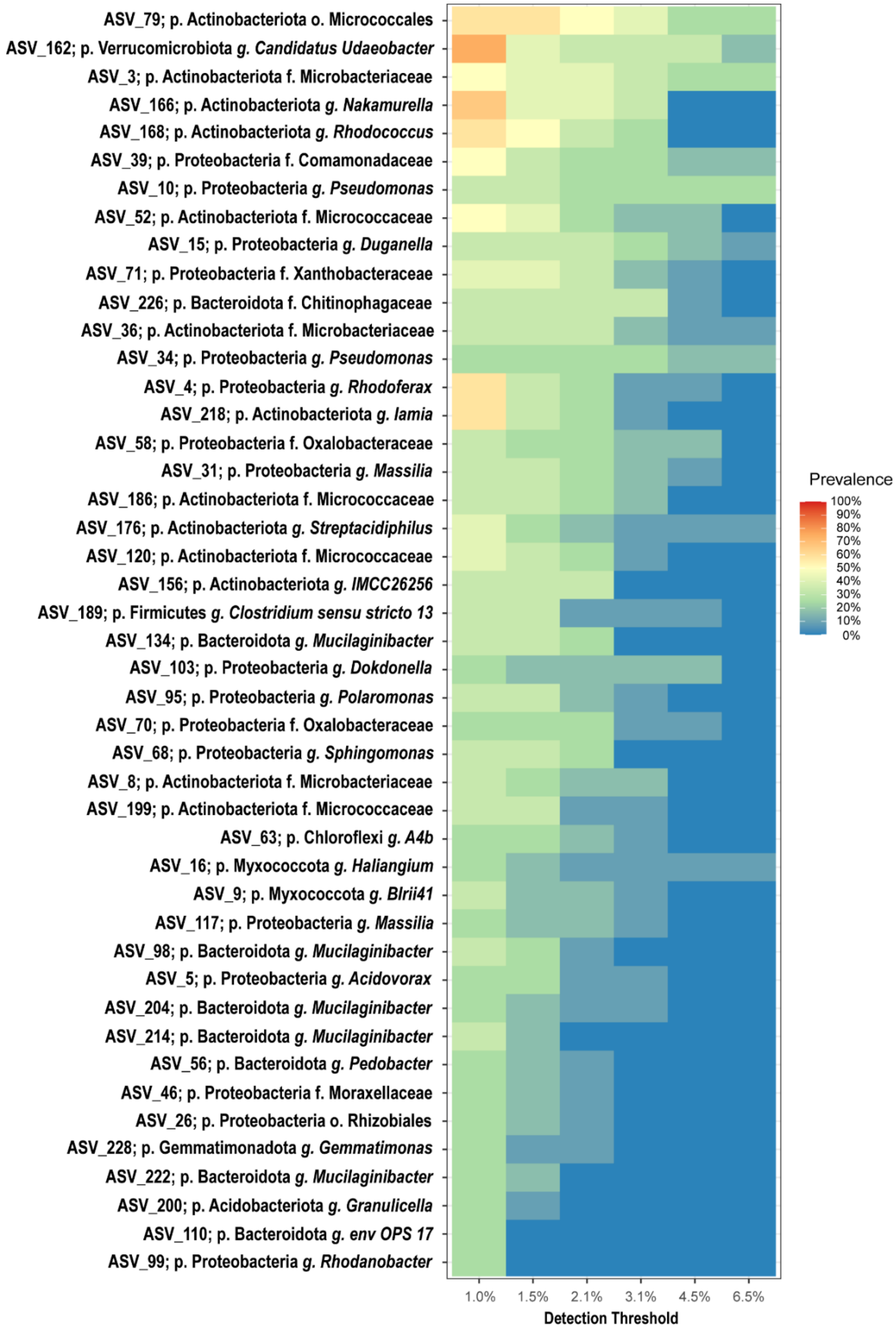

Supplementary Figure S5. Heatmap of zOTUS prevalence across detection thresholds, illustrating the relative distribution of bacterial genera according to their frequency of occurrence in Coppermine Cove.

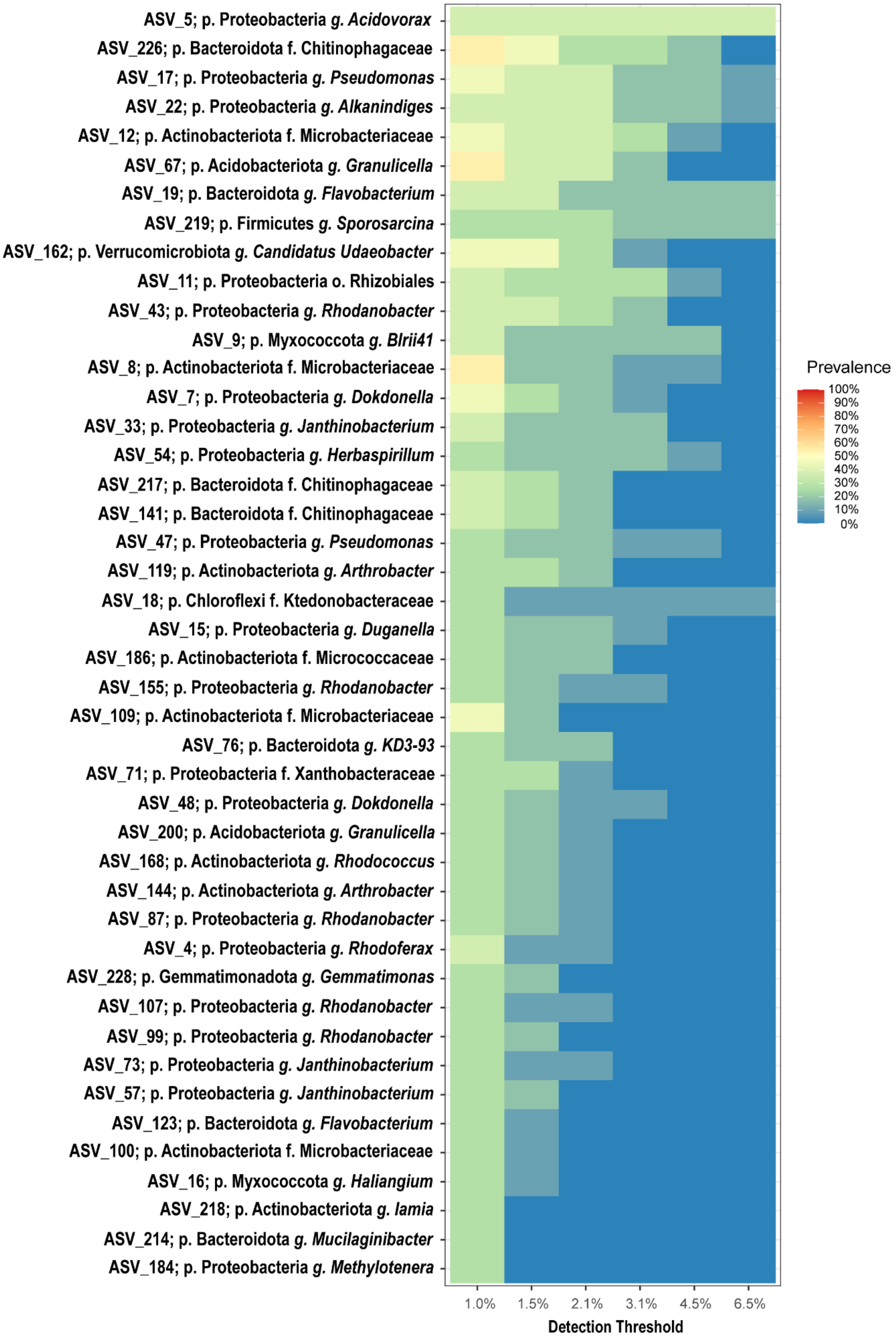

Supplementary Figure S6. Heatmap of zOTUS prevalence across detection thresholds, illustrating the relative distribution of bacterial genera according to their occurrence frequency in Byers Peninsula.

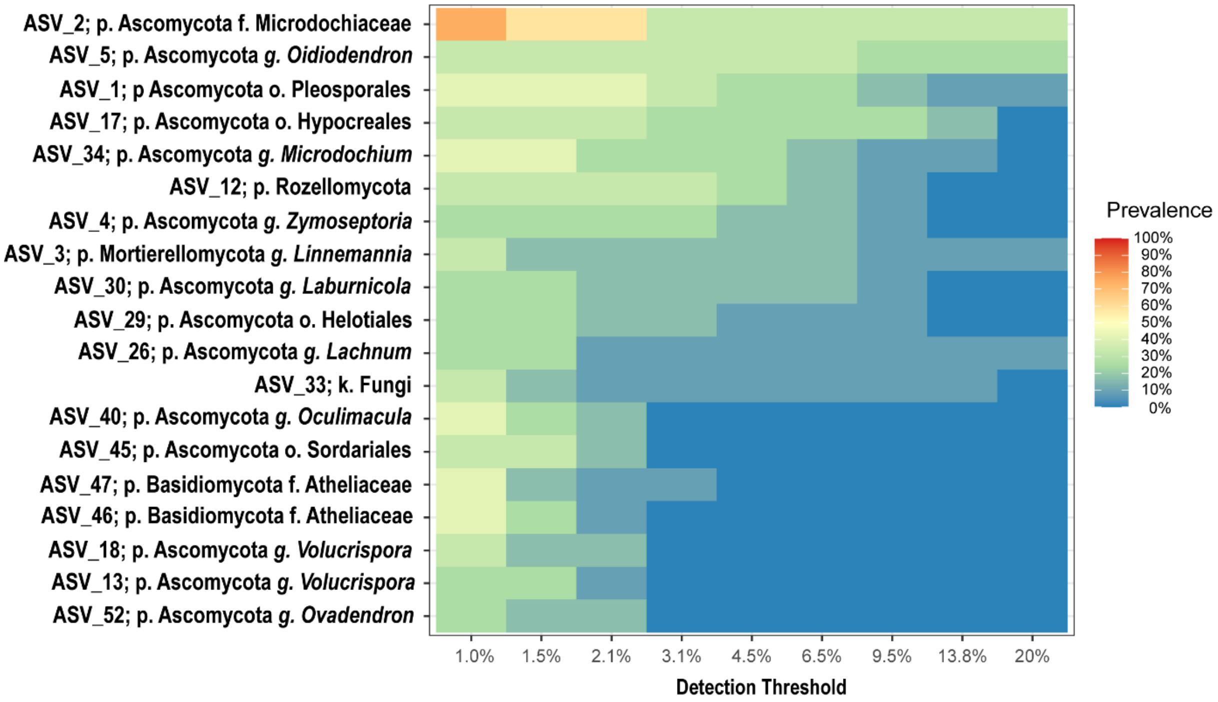

Supplementary Figure S7. Heatmap of zOTUS prevalence across detection thresholds, illustrating the relative distribution of Fungal genera according to their occurrence frequency in Byers Peninsula.

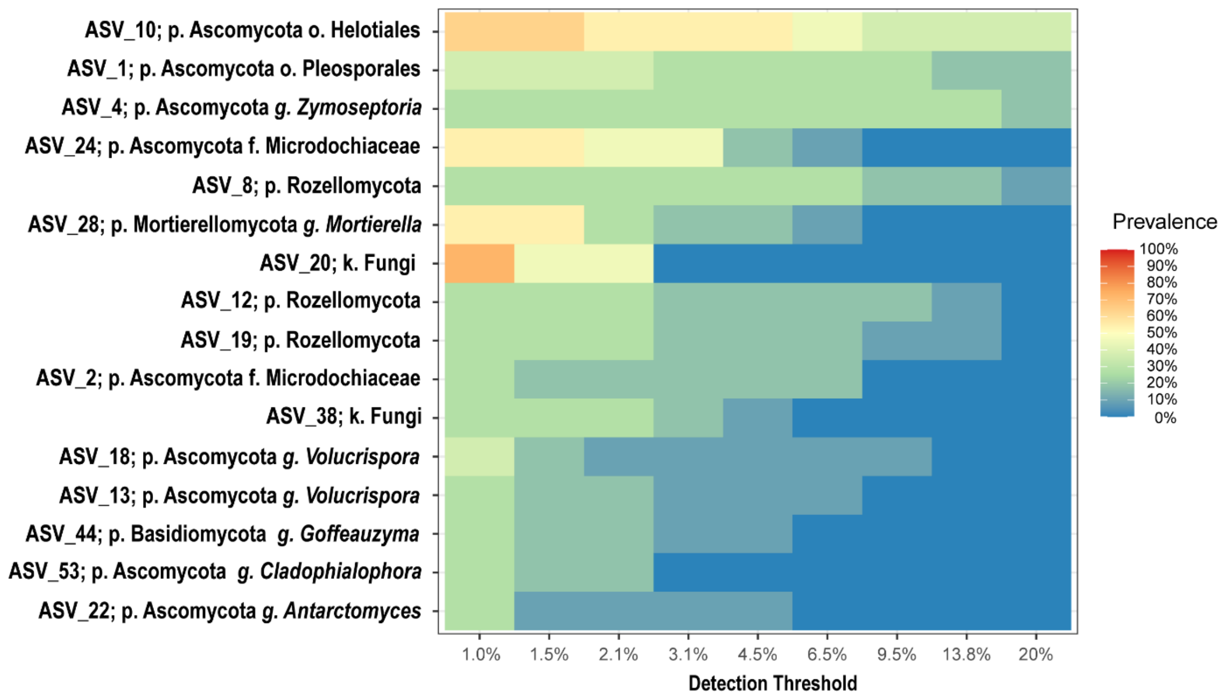

Supplementary Figure S8. Heatmap of zOTUS prevalence across detection thresholds, illustrating the relative distribution of Fungal genera according to their occurrence frequency in Aldmiralty bay.

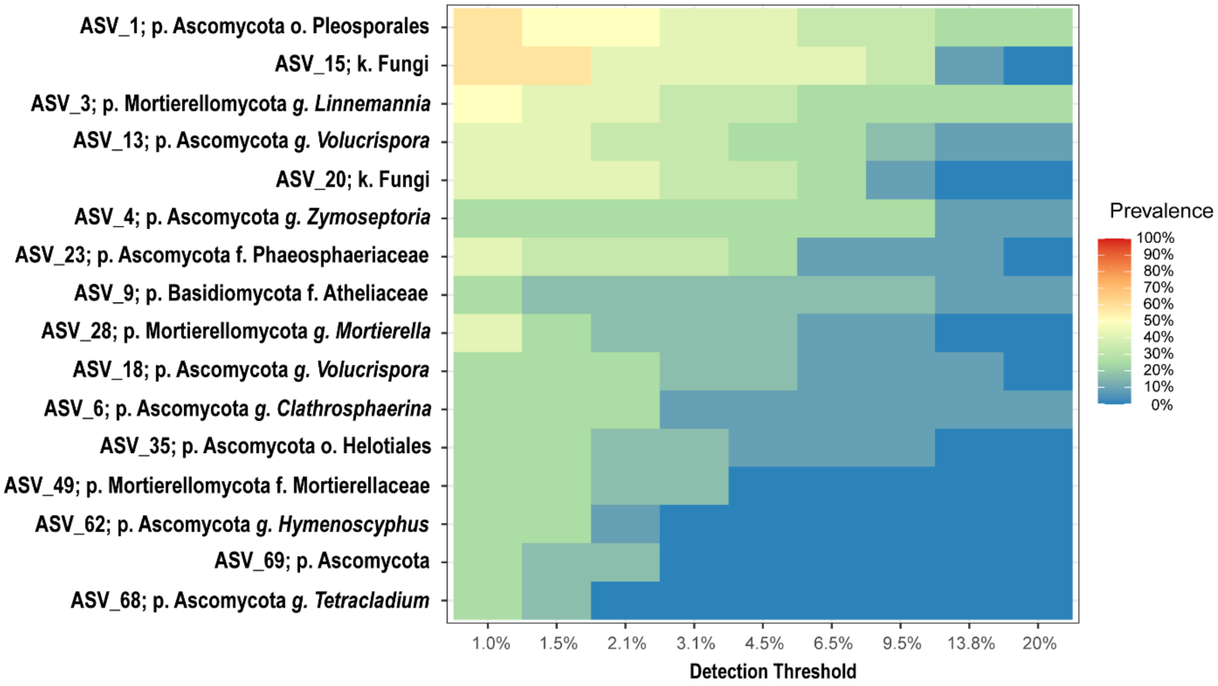

Supplementary Figure S9. Heatmap of zOTUS prevalence across detection thresholds, illustrating the relative distribution of Fungal genera according to their occurrence frequency in Coppermine Cove.

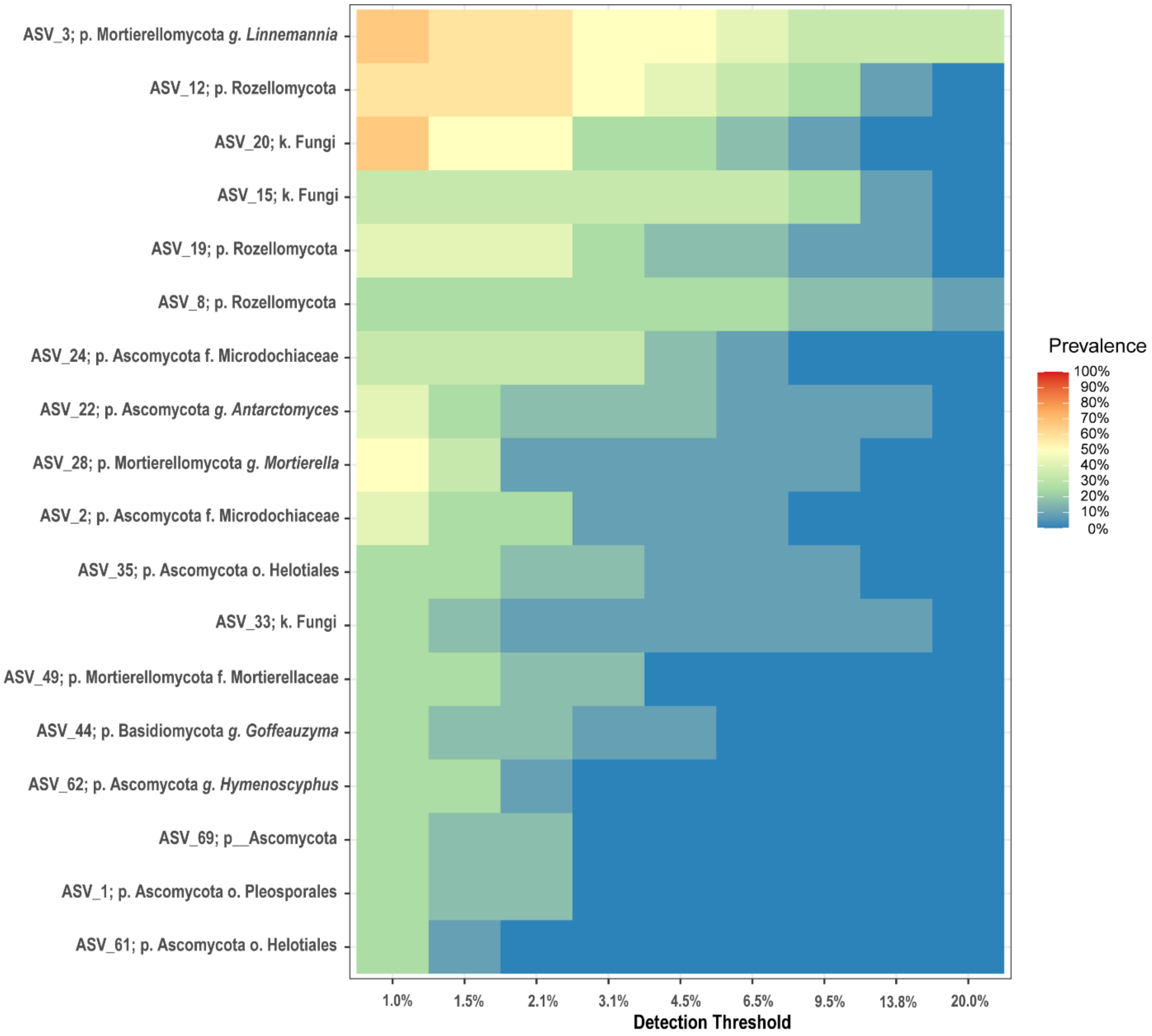

Supplementary Figure S10. Heatmap of zOTUS prevalence across detection thresholds, illustrating the relative distribution of Fungal genera according to their occurrence frequency in Rhizosphere.

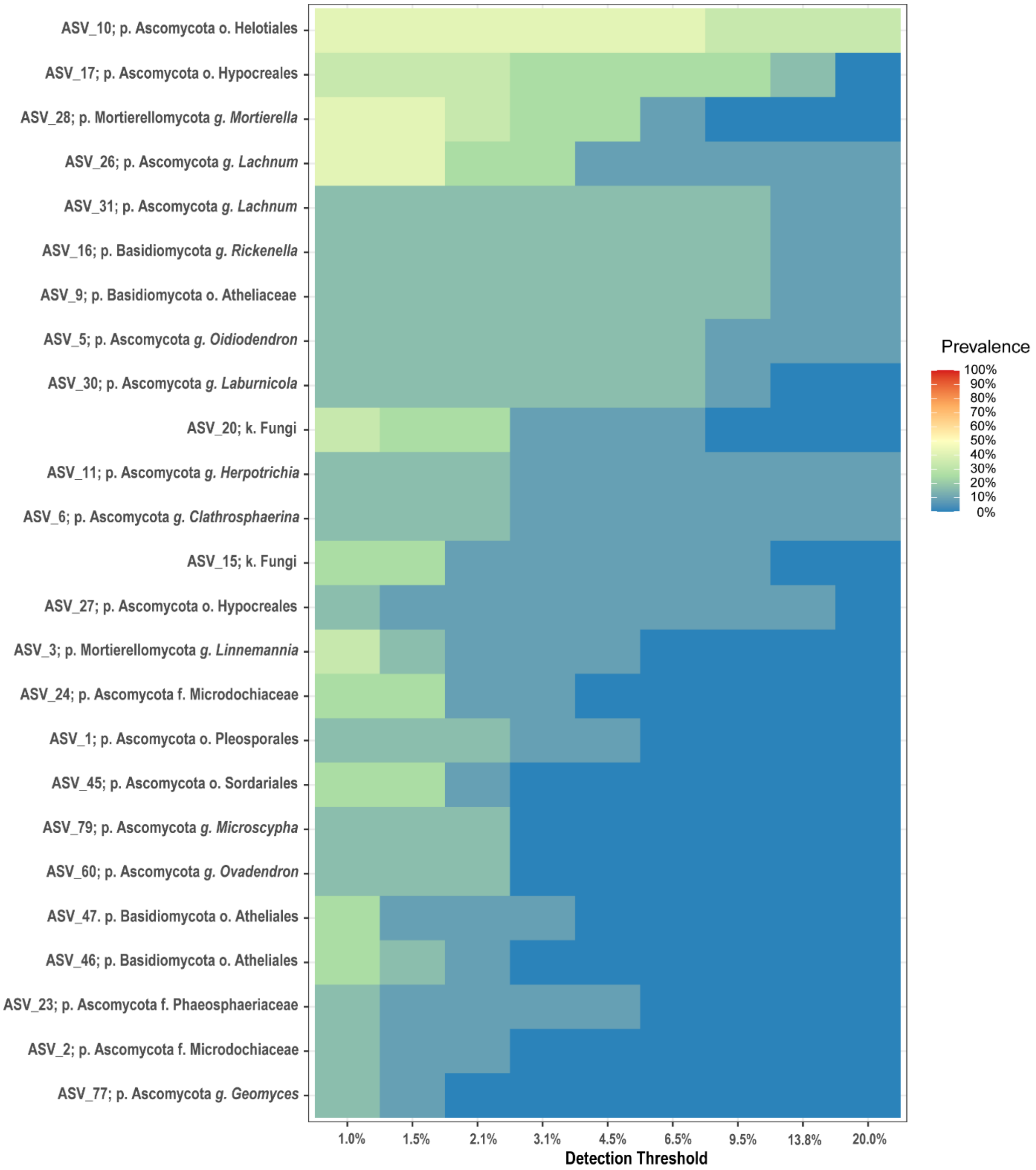

Supplementary Figure S11. Heatmap of Fungal zOTUS prevalence across detection thresholds, illustrating the relative distribution of Fungal genera according to their occurrence frequency in Root Endosphere.

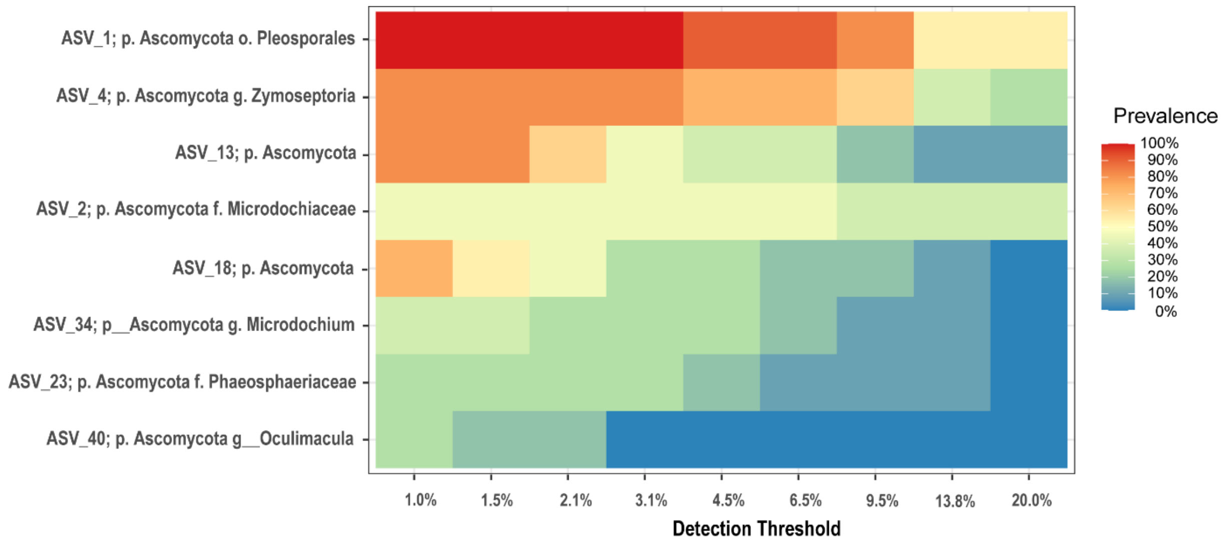

Supplementary Figure S12. Heatmap of Fungal zOTUS prevalence across detection thresholds, illustrating the relative distribution of Fungal genera according to their occurrence frequency in Leaf endosphere.

FUNGI GUILD

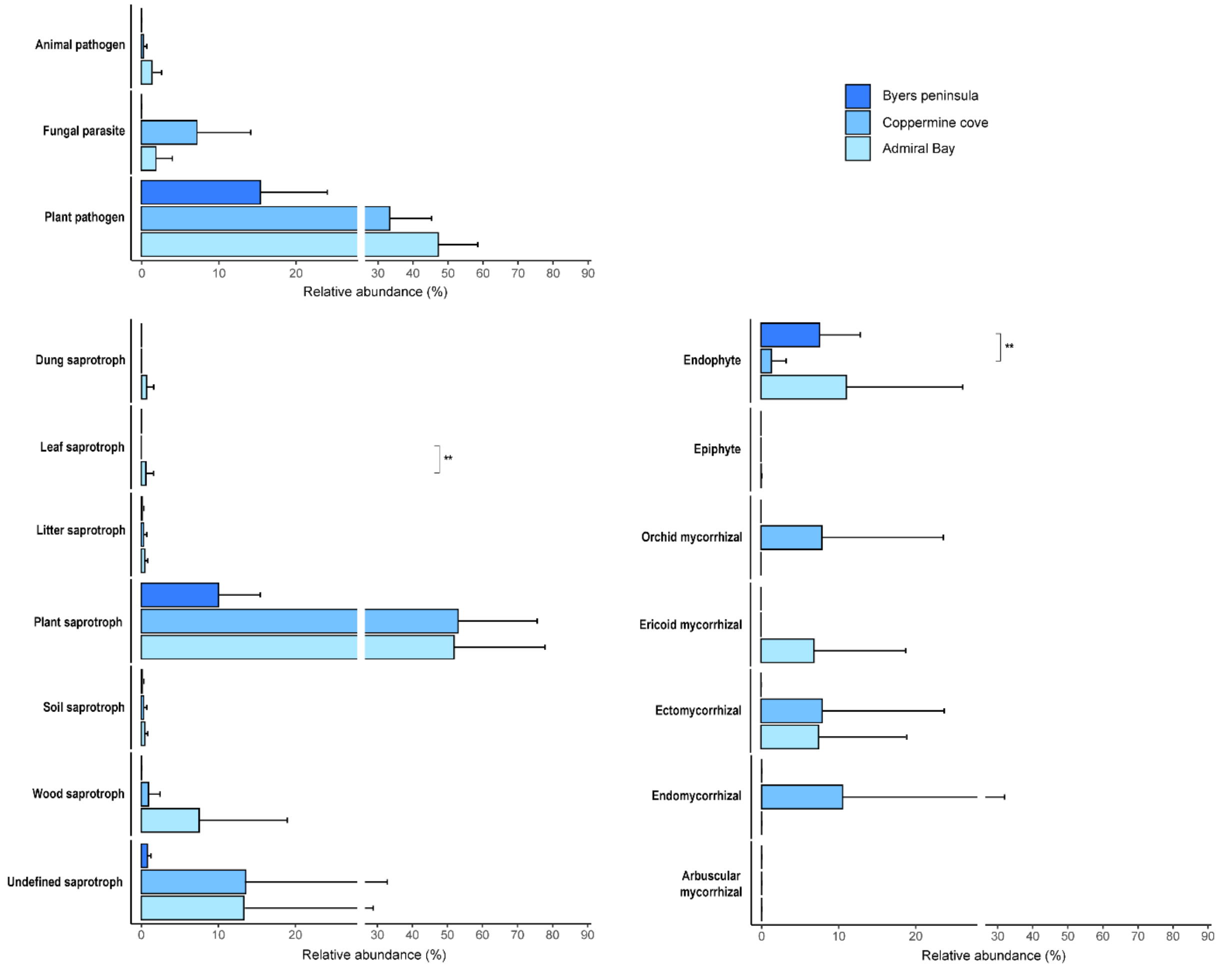

Supplementary Figure S13. Relative abundance of fungal functional guilds (FungiGuild) of Leaf endosphere across Byers Peninsula, Coppermine Cove, and Admiralty Bay, classified by ecological roles including pathogens, saprotrophs, and symbiotrophs.

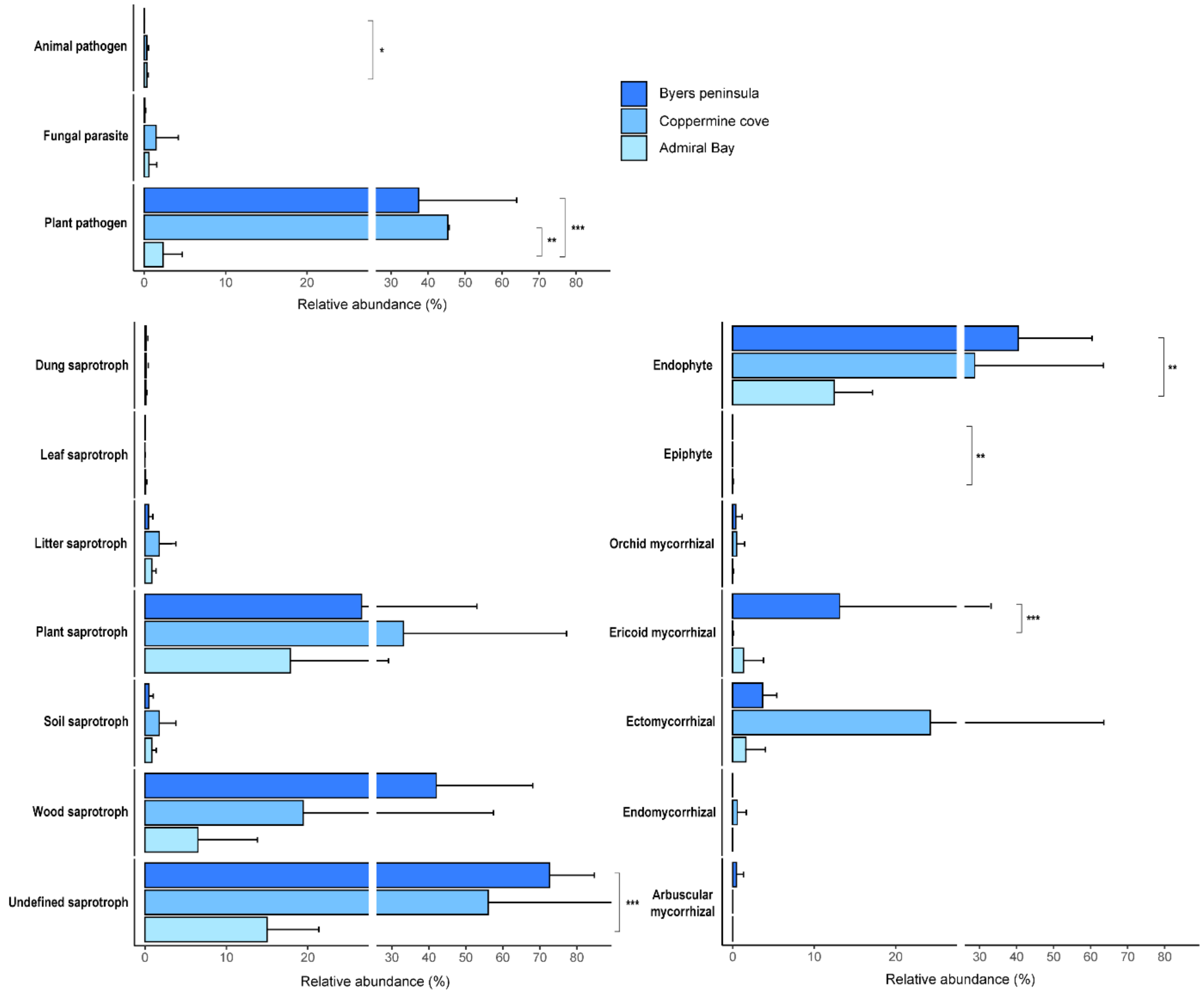

Supplementary Figure S14. Relative abundance of fungal functional guilds (FungiGuild) of Root endosphere across Byers Peninsula, Coppermine Cove, and Admiralty Bay, classified by ecological roles including pathogens, saprotrophs, and symbiotrophs.

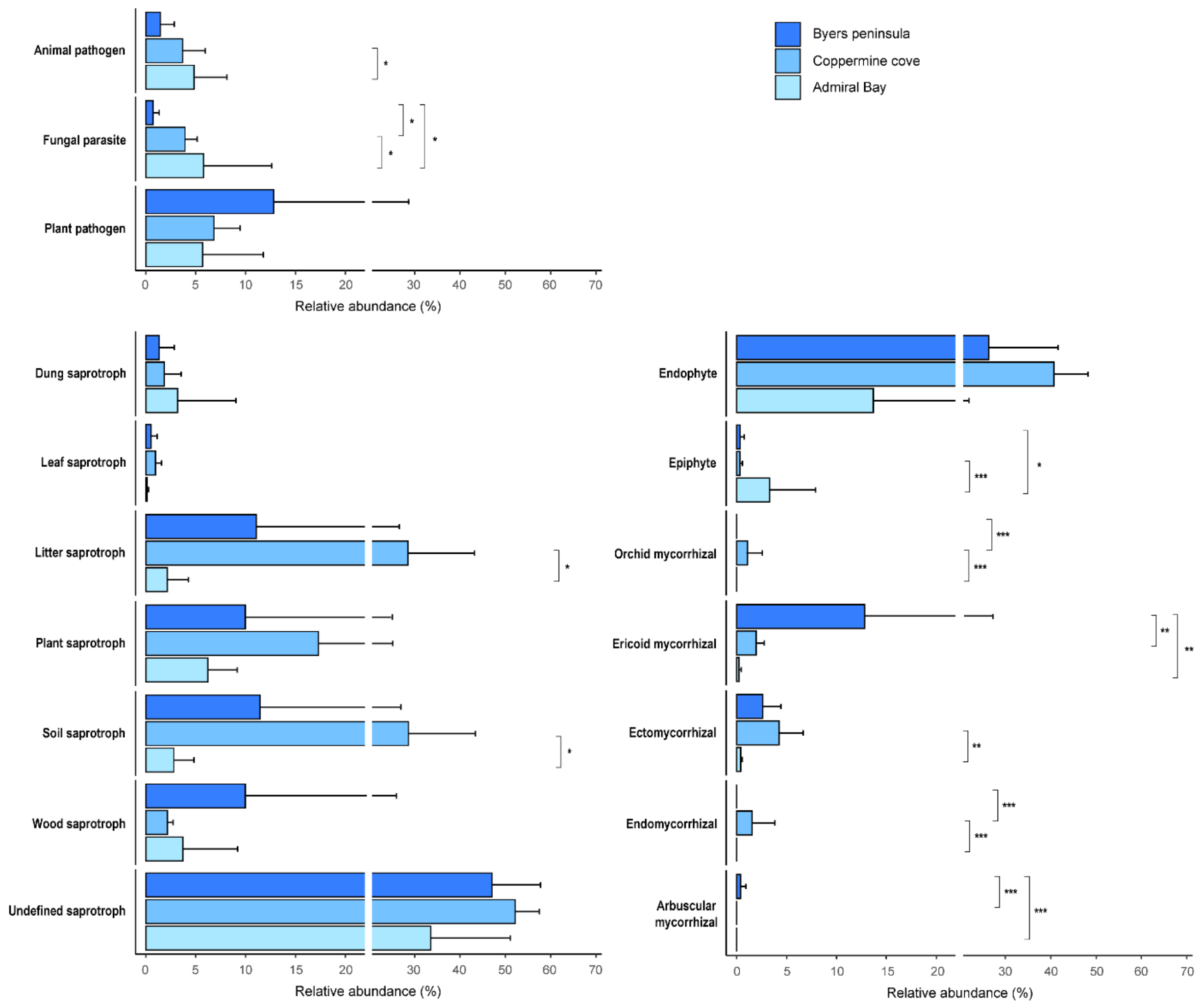

Supplementary Figure S15. Relative abundance of fungal functional guilds (FungiGuild) of Rhizosphereacross Byers Peninsula, Coppermine Cove, and Admiralty Bay, classified by ecological roles including pathogens, saprotrophs, and symbiotrophs.

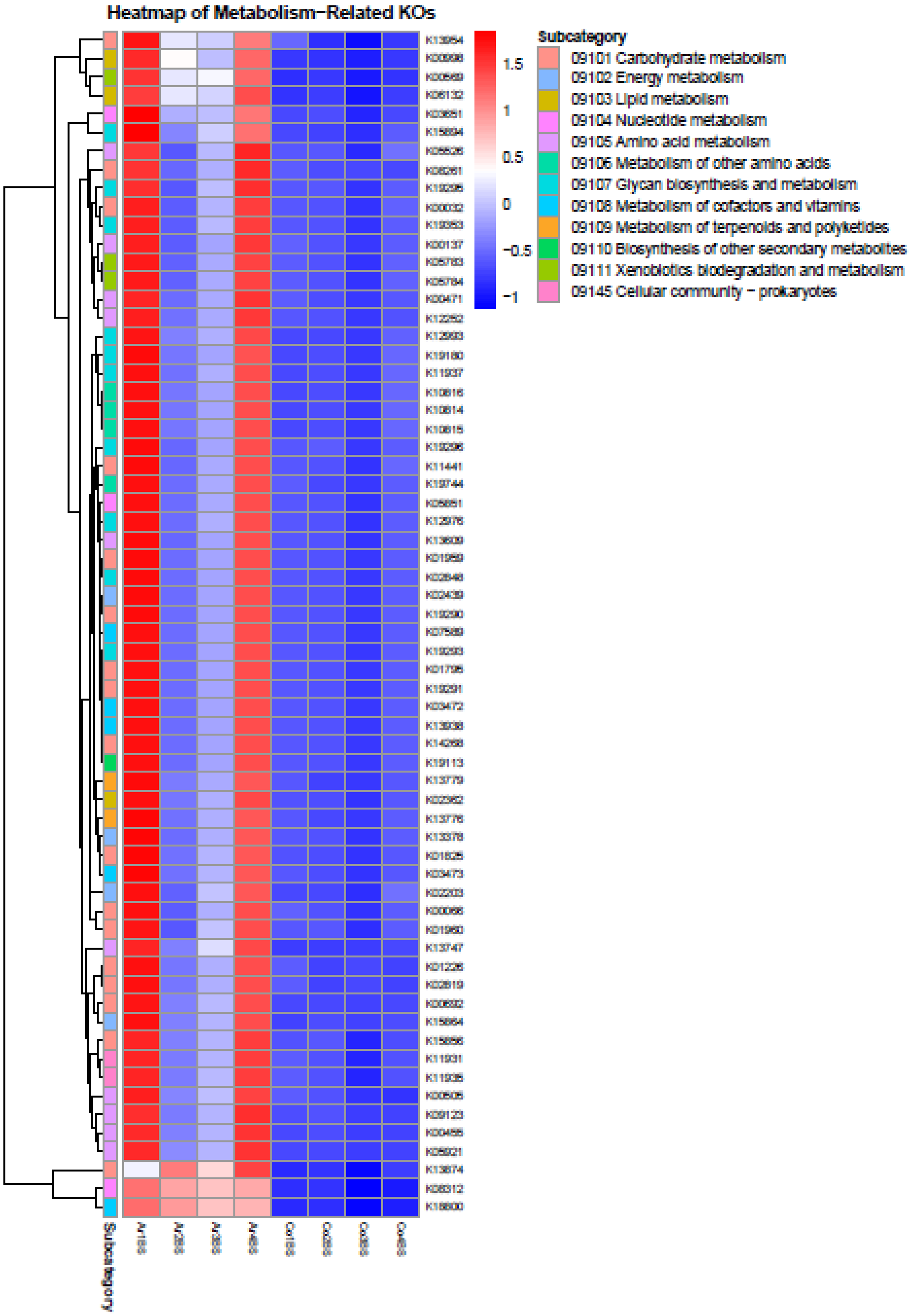

Supplementary Figure S16. Heatmap of metabolism-related KEGG Orthologs in rhizosphere samples from Admiralty Bay and Coppermine Cove.

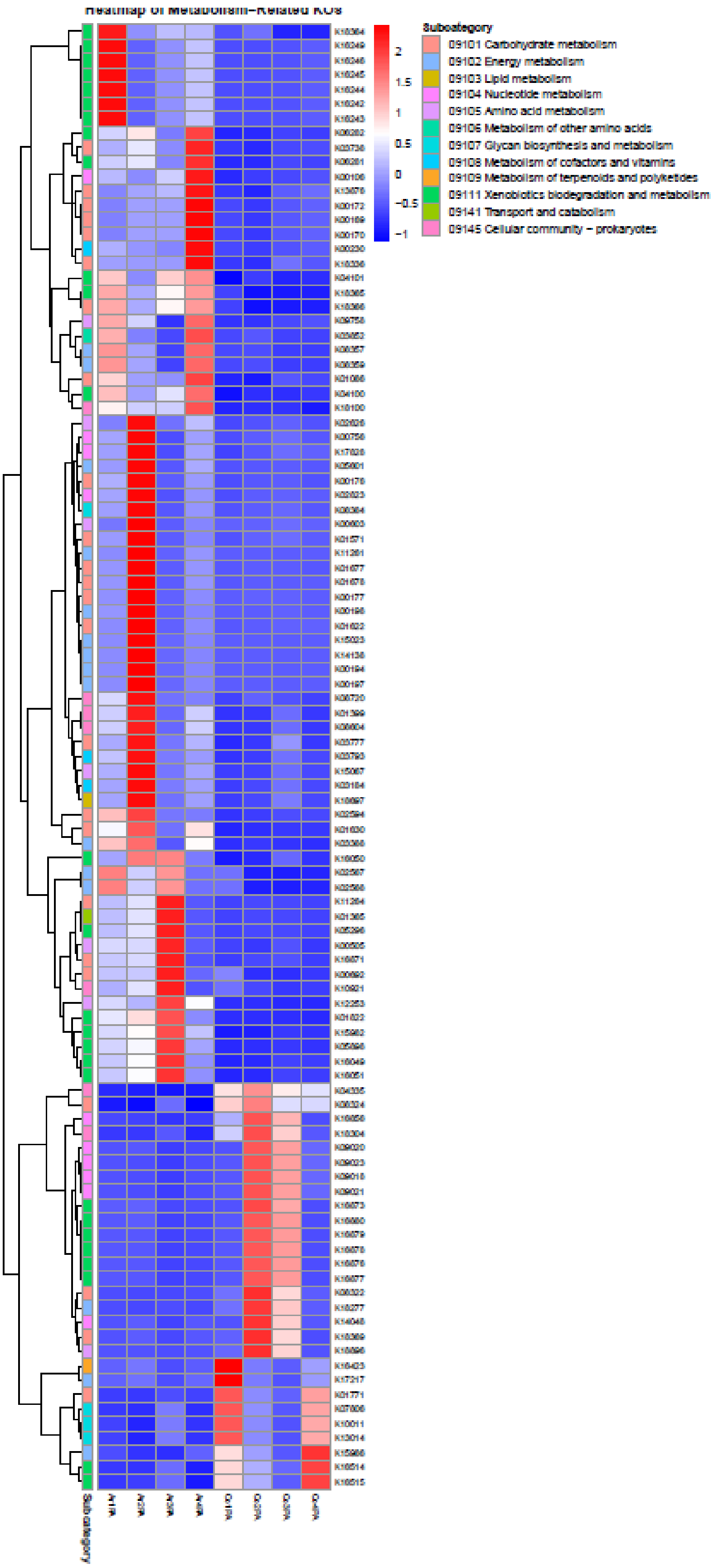

Supplementary Figure S17. Heatmap of metabolism-related KEGG Orthologs in leaf endosphere samples from Admiralty Bay and Coppermine Cove.

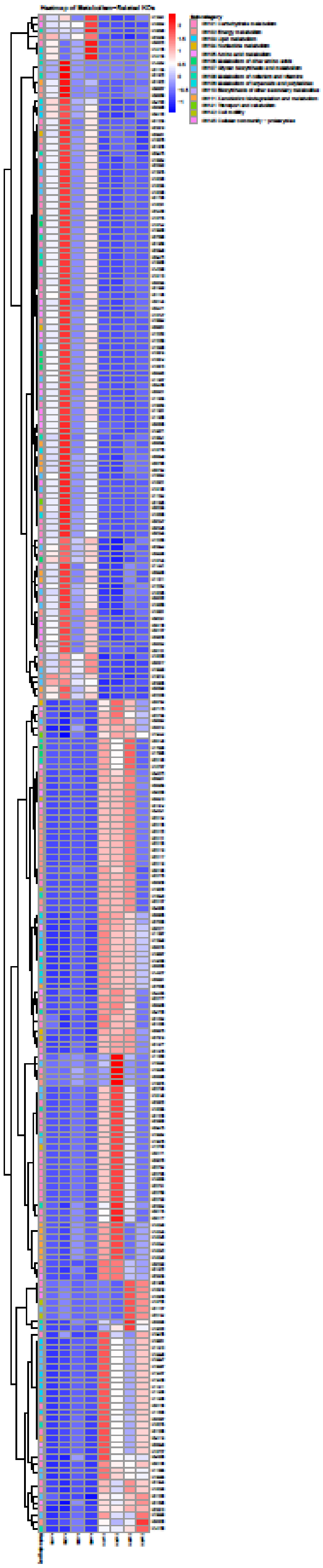

Supplementary Figure S18. Heatmap of metabolism-related KEGG Orthologs in root endosphere samples from Admiralty Bay and Coppermine Cove.

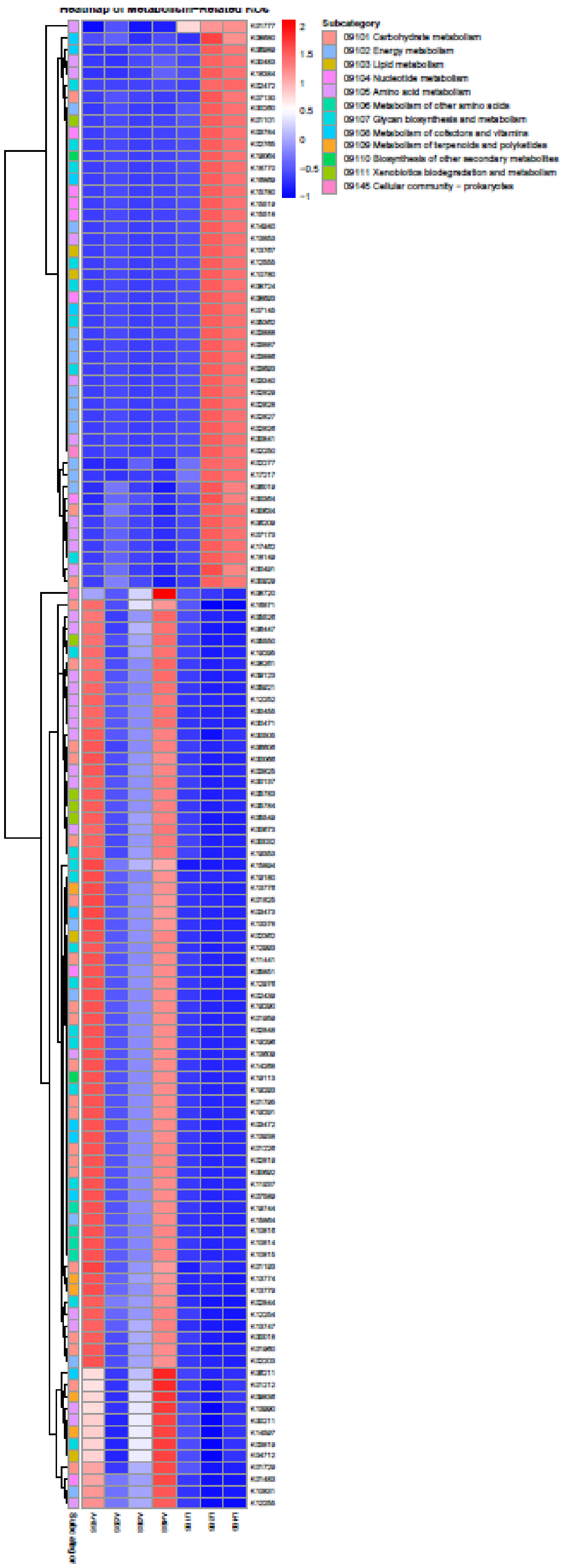

Supplementary Figure S19. Heatmap of metabolism-related KEGG Orthologs in rhizosphere samples from Admiralty Bay and Byers Peninsula.

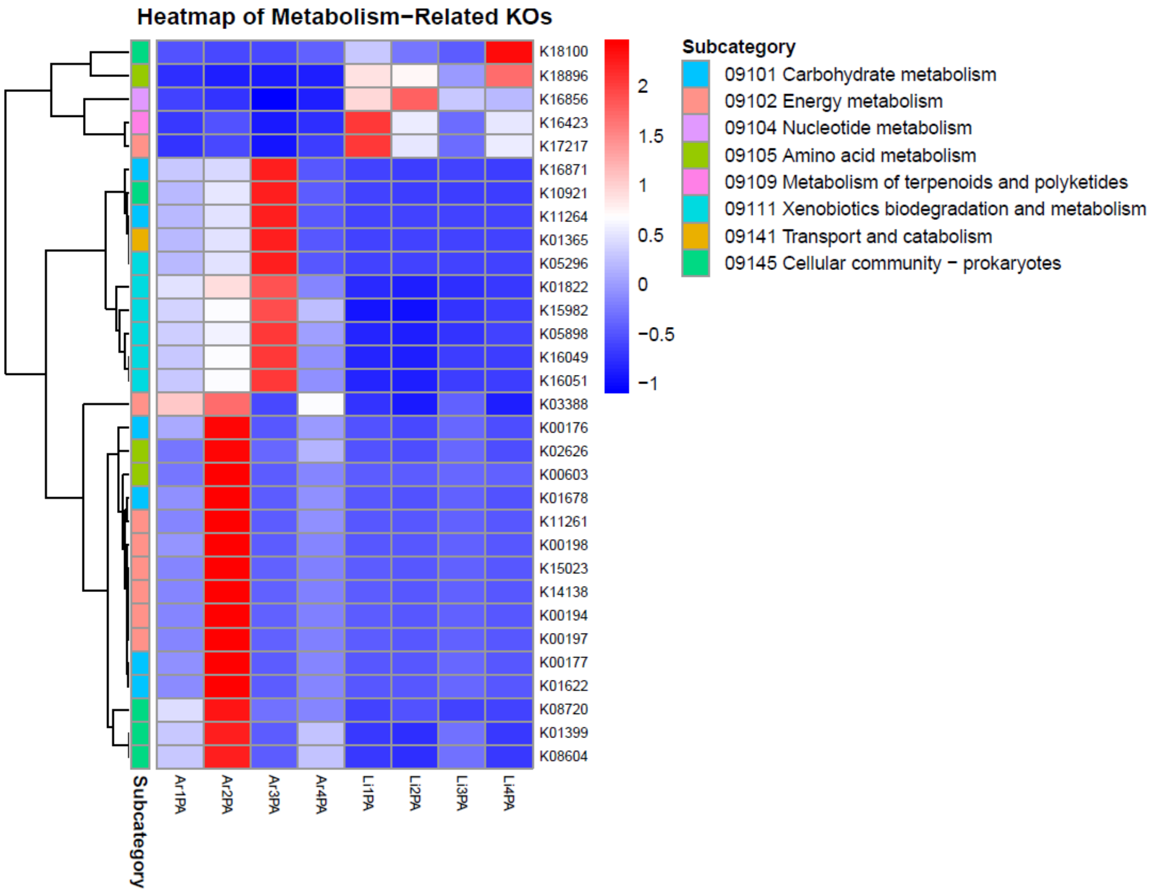

Supplementary Figure S20. Heatmap of metabolism-related KEGG Orthologs in leaf endosphere samples from Admiralty Bay and Byers Peninsula.

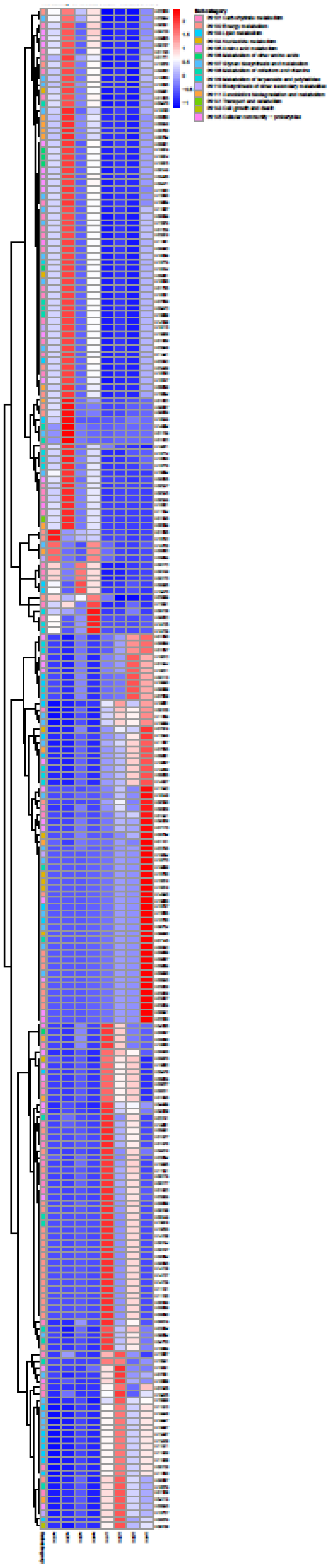

Supplementary Figure S21. Heatmap of metabolism-related KEGG Orthologs in root endosphere samples from Admiralty Bay and Byers Peninsula.

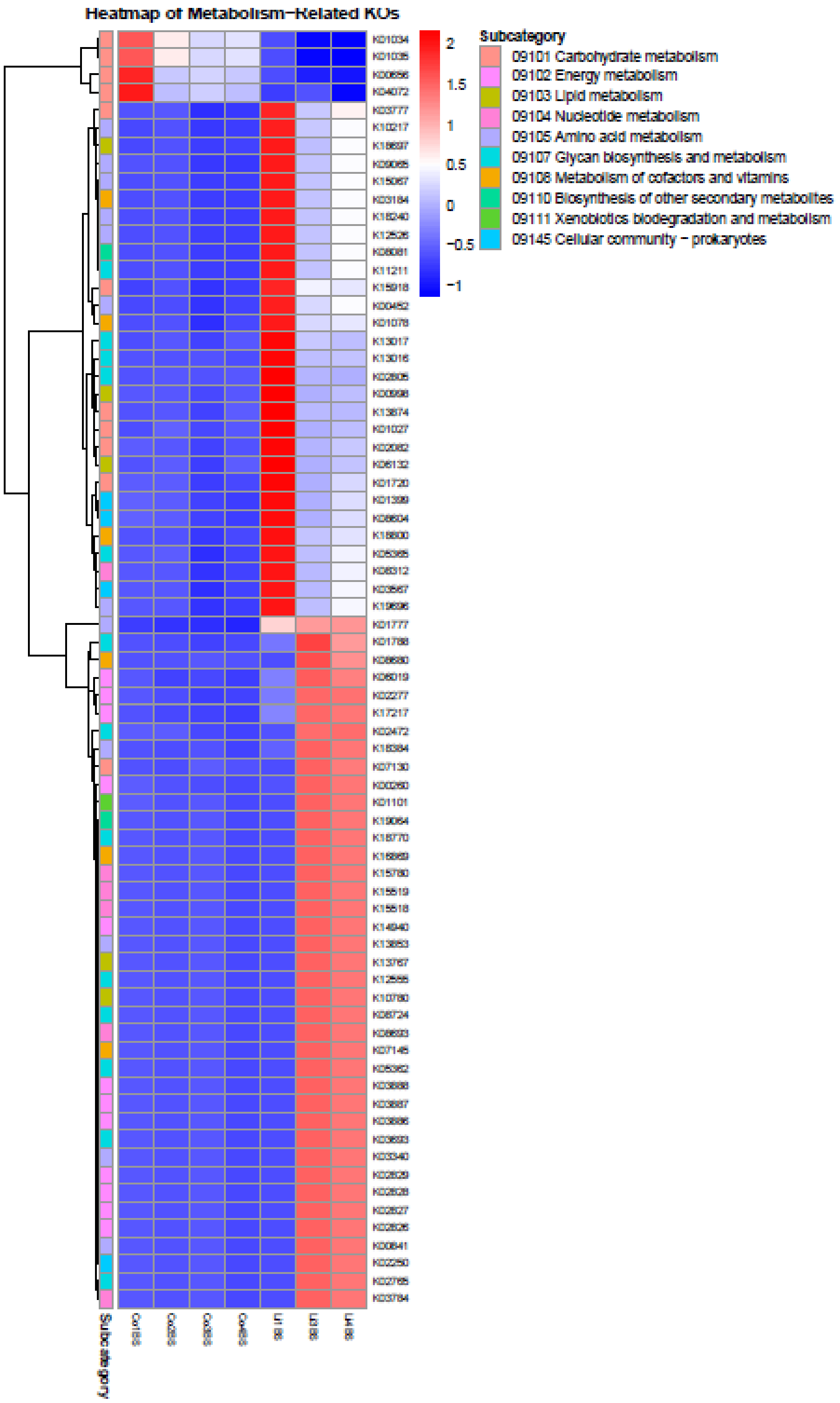

Supplementary Figure S22. Heatmap of metabolism-related KEGG Orthologs in rhizosphere samples from Coppermine Cove and Byers Peninsula.

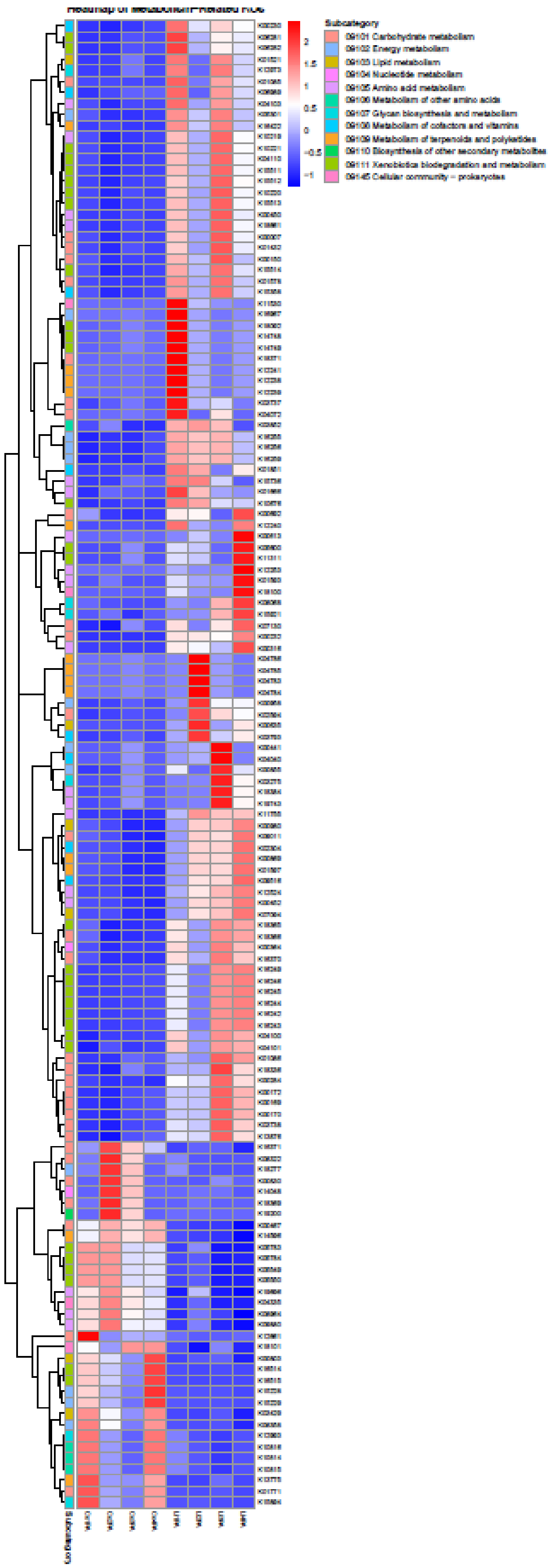

Supplementary Figure S23. Heatmap of metabolism-related KEGG Orthologs in leaf endosphere samples from Coppermine Cove and Byers Peninsula.

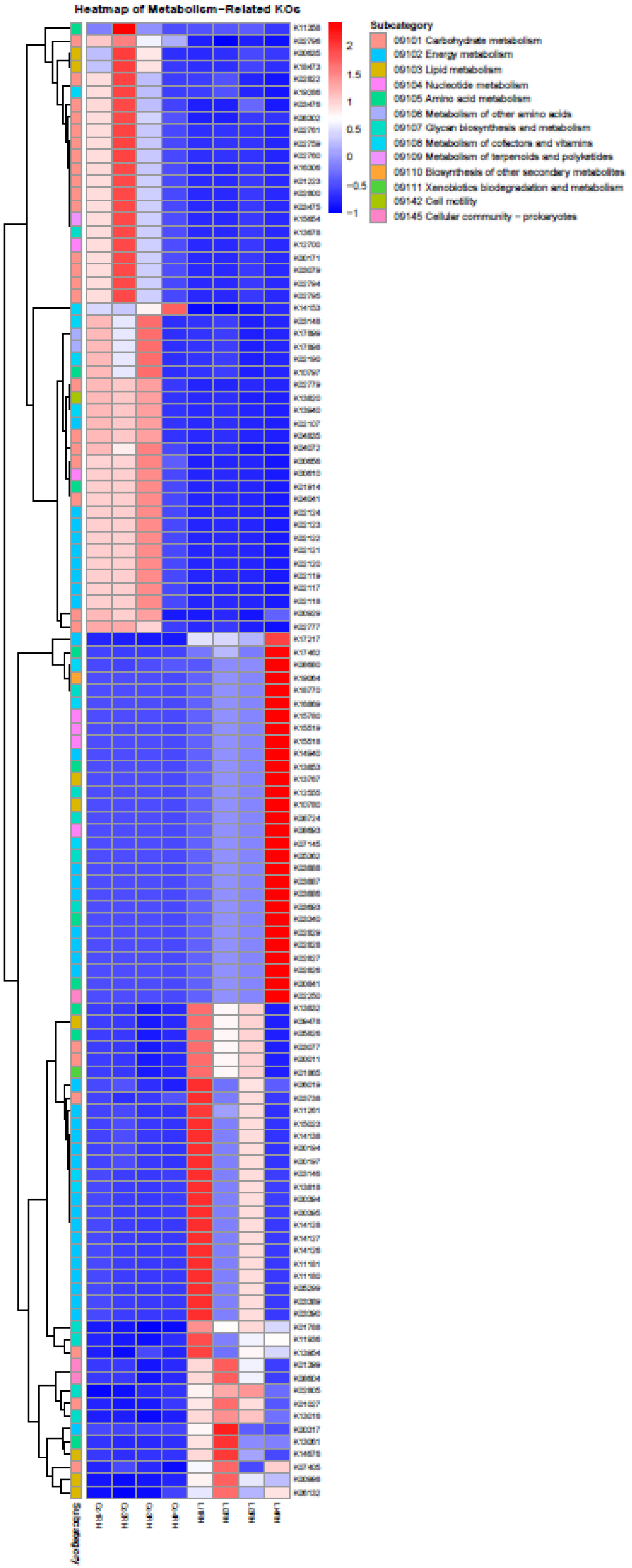

Supplementary Figure S24. Heatmap of metabolism-related KEGG Orthologs in root endosphere samples from Coppermine Cove and Byers Peninsula.

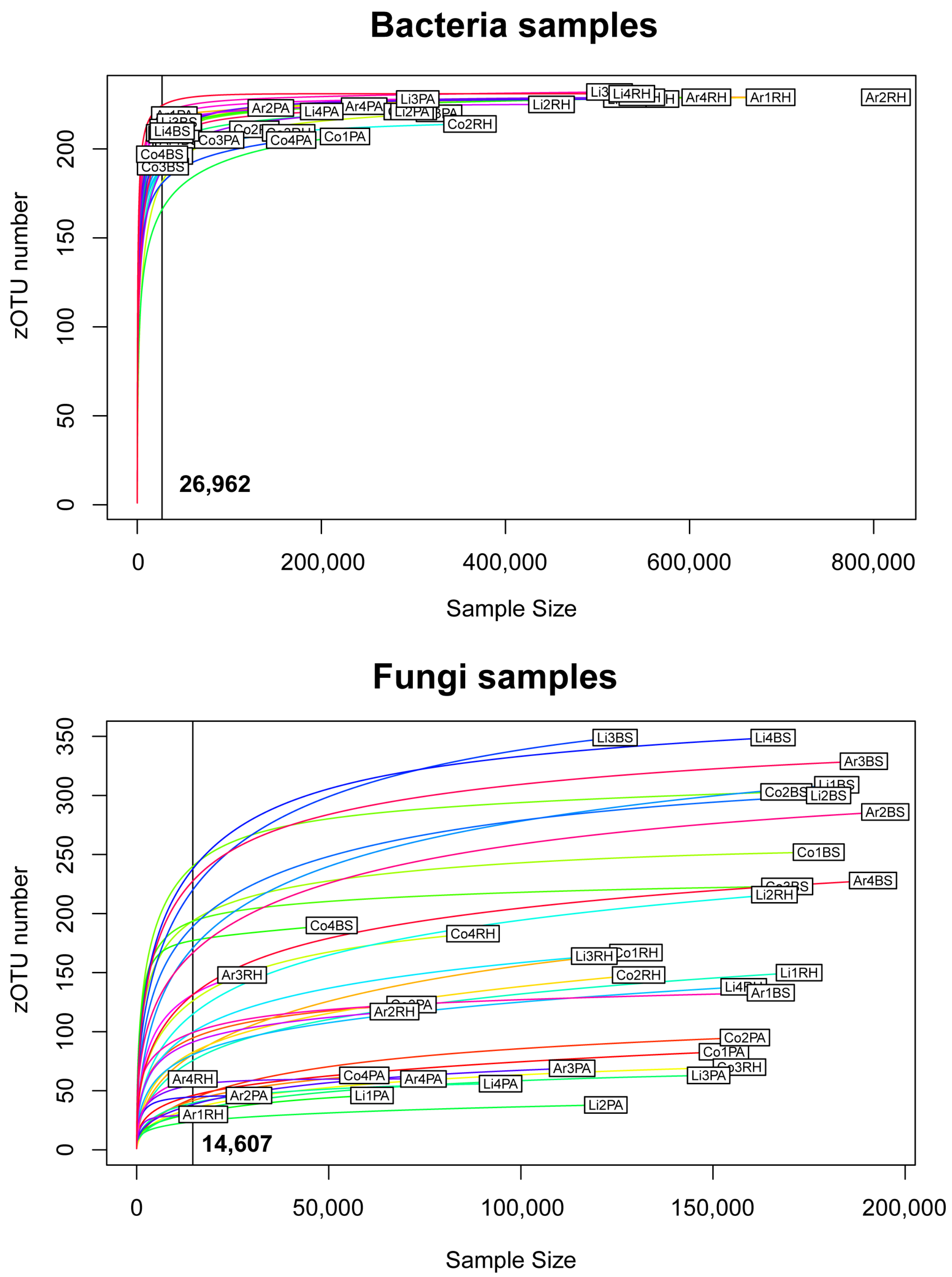

Supplementary Figure S25. Rarefaction curves and sample retention summary for bacterial (16S rRNA) and fungal (ITS1) datasets. Curves show sequencing depth versus observed features (zOTUs) after quality filtering. Vertical dashed lines indicate the selected rarefaction depths used for alpha diversity analyses (26,962 for bacteria; 14,607 for fungi). All samples retained sufficient sequencing depth for robust diversity estimation.

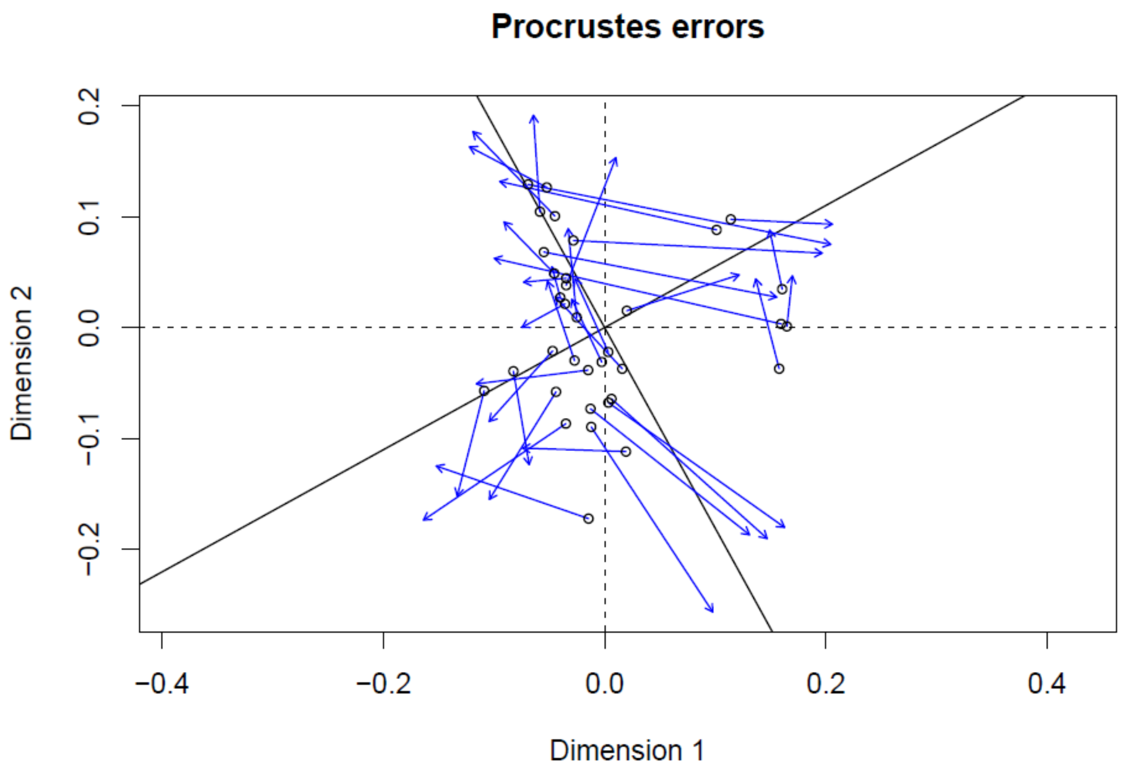

Supplementary Figure S26. Procrustes and Mantel analyses showing the correlation between microbial community composition and predicted functional profiles inferred by PICRUSt2. (A) Procrustes ordination displaying concordance between Aitchison distance matrices derived from CLR-transformed taxonomic and functional datasets. (B) Mantel test results (r = 0.451, p = 0.001) indicating a significant correlation between community structure and inferred functional repertoires.

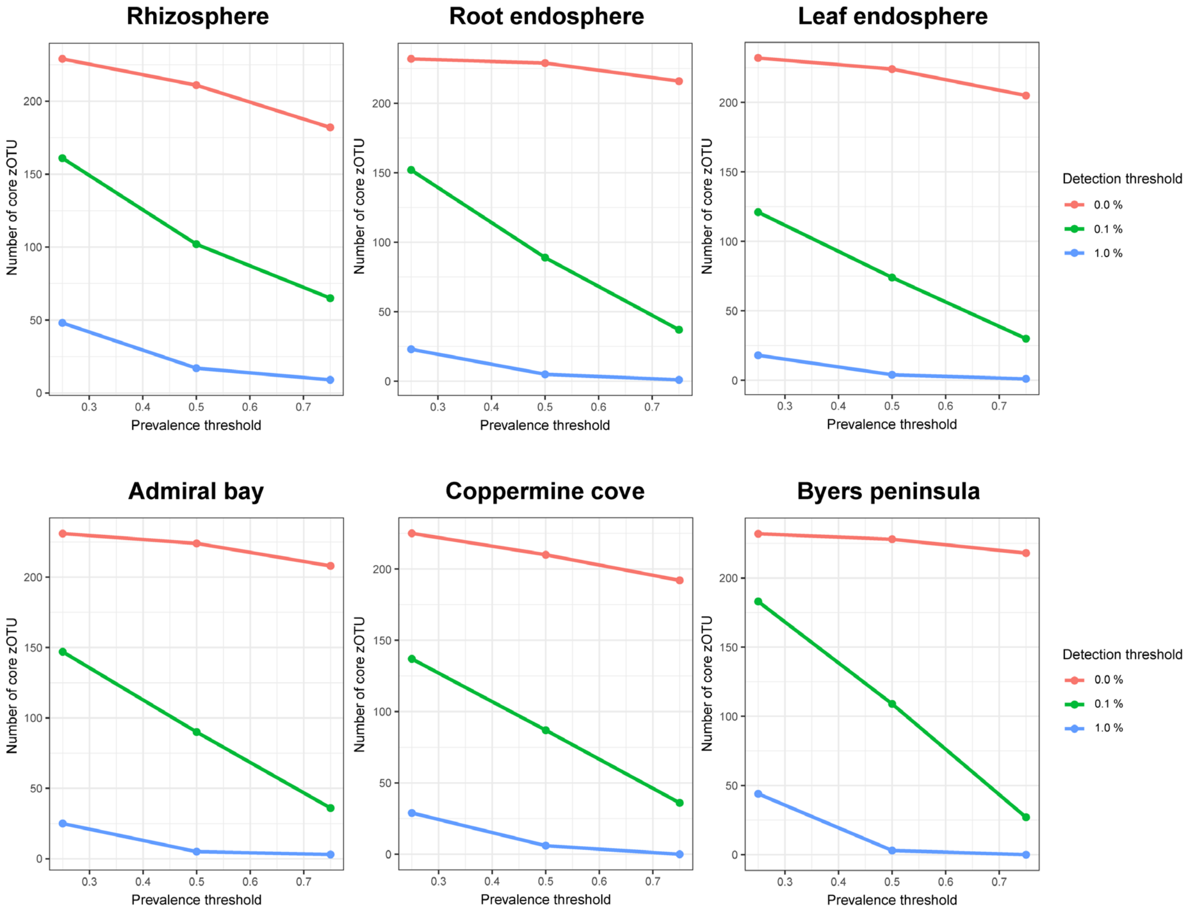

Supplementary Figure S27. Sensitivity analysis of bacterial core microbiomes under varying detection thresholds. The number of core taxa (y-axis) is shown across prevalence thresholds (25%, 50%, 75%) and minimum relative abundance cutoffs (0%, 0.1%, 1%). Core size decreased smoothly with increasing stringency, confirming the robustness of the identified bacterial core.

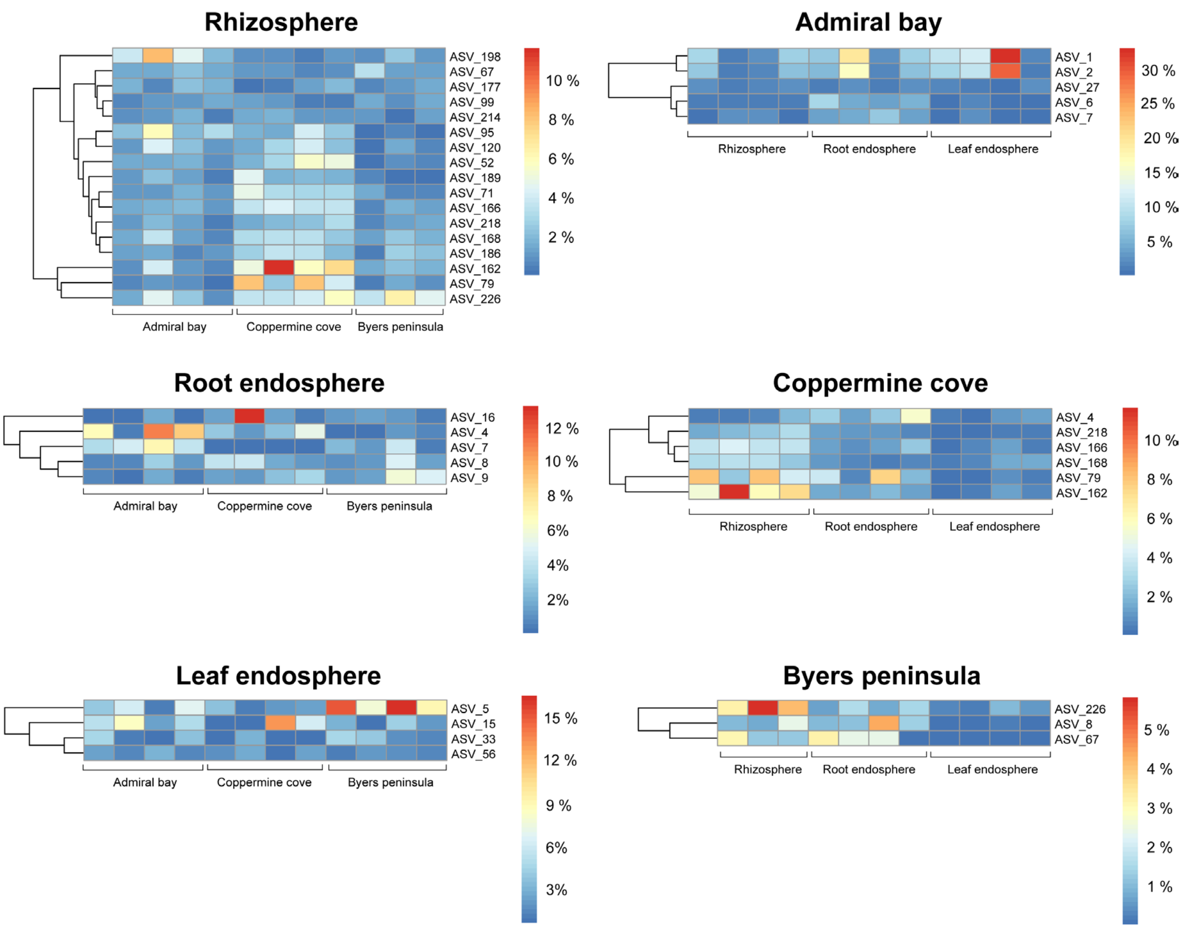

Supplementary Figure S28. Heatmap of relative abundances of bacterial core taxa across plant compartments (rhizosphere, root endosphere, leaf endosphere) and sampling sites (Admiralty Bay, Coppermine Cove, Byers Peninsula). Only taxa meeting the ≥50% prevalence and ≥1% relative abundance thresholds are displayed. Color intensity represents relative abundance after CLR transformation.

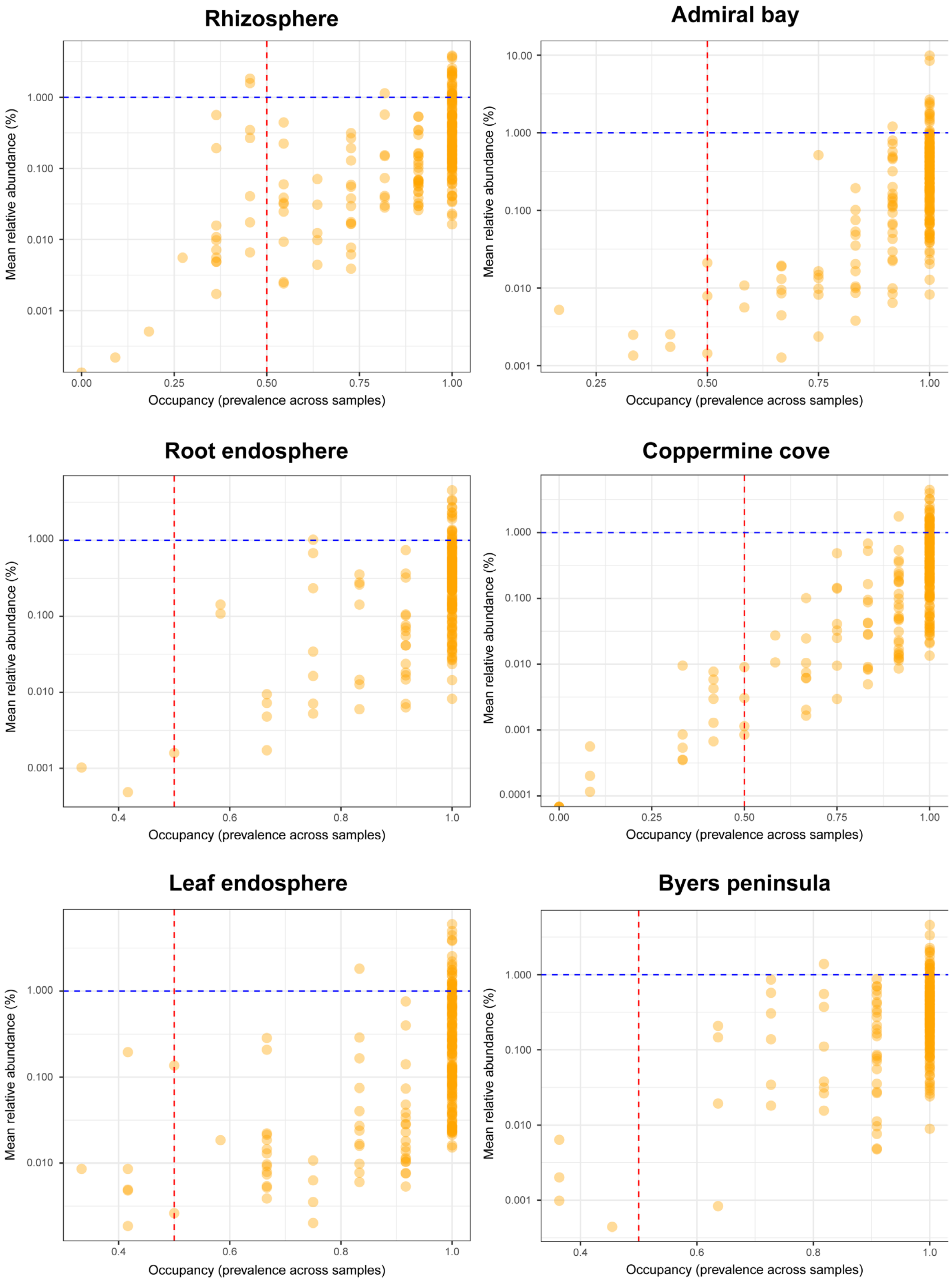

Supplementary Figure S29. Occupancy–abundance plot for bacterial taxa across all samples. Each point represents a zOTU colored by phylum-level classification. Core taxa occupy the upper right quadrant (high occupancy, high abundance), indicating their consistent presence and dominance across compartments and islands.

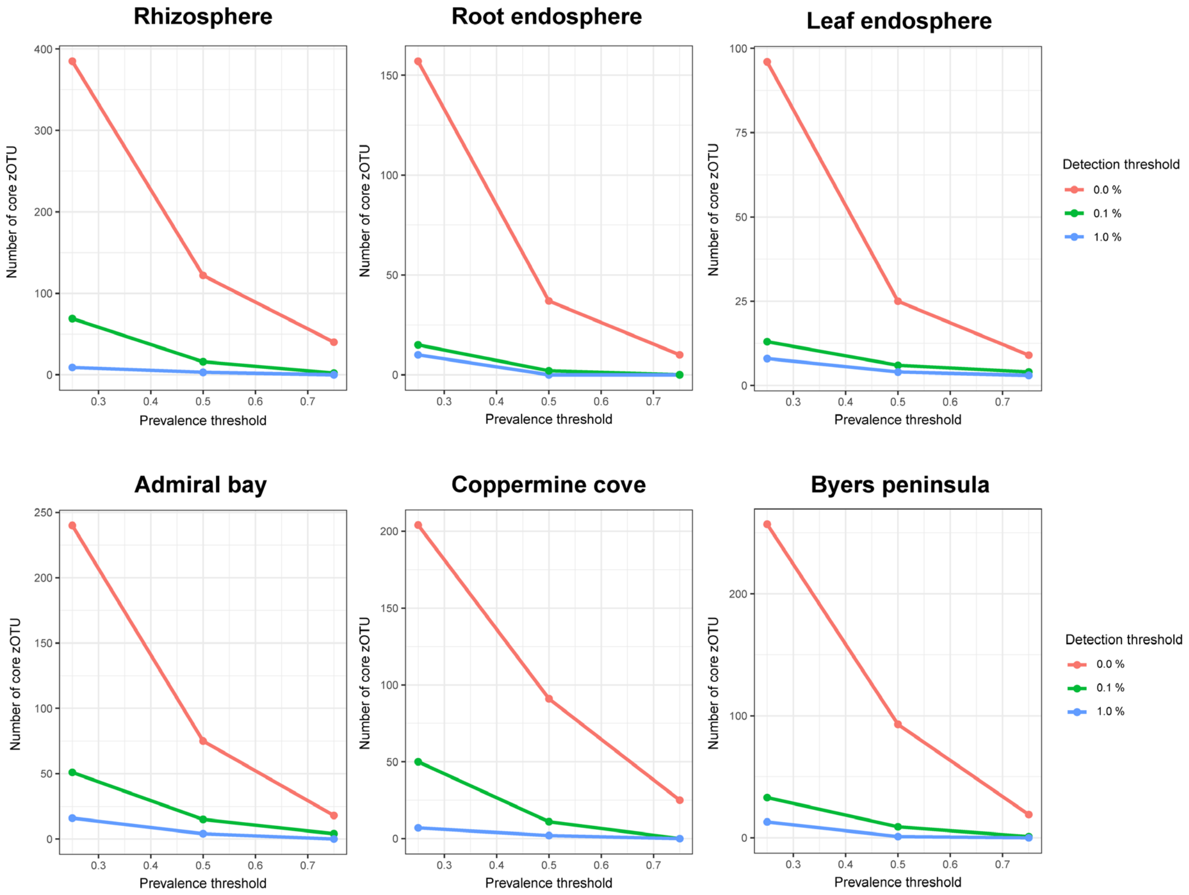

Supplementary Figure S30. Sensitivity analysis of fungal core microbiomes under varying detection thresholds. The number of core taxa (y-axis) is shown across prevalence thresholds (25%, 50%, 75%) and minimum relative abundance cutoffs (0%, 0.1%, 1%). Core fungal composition remained stable across thresholds, confirming the robustness of core definitions.

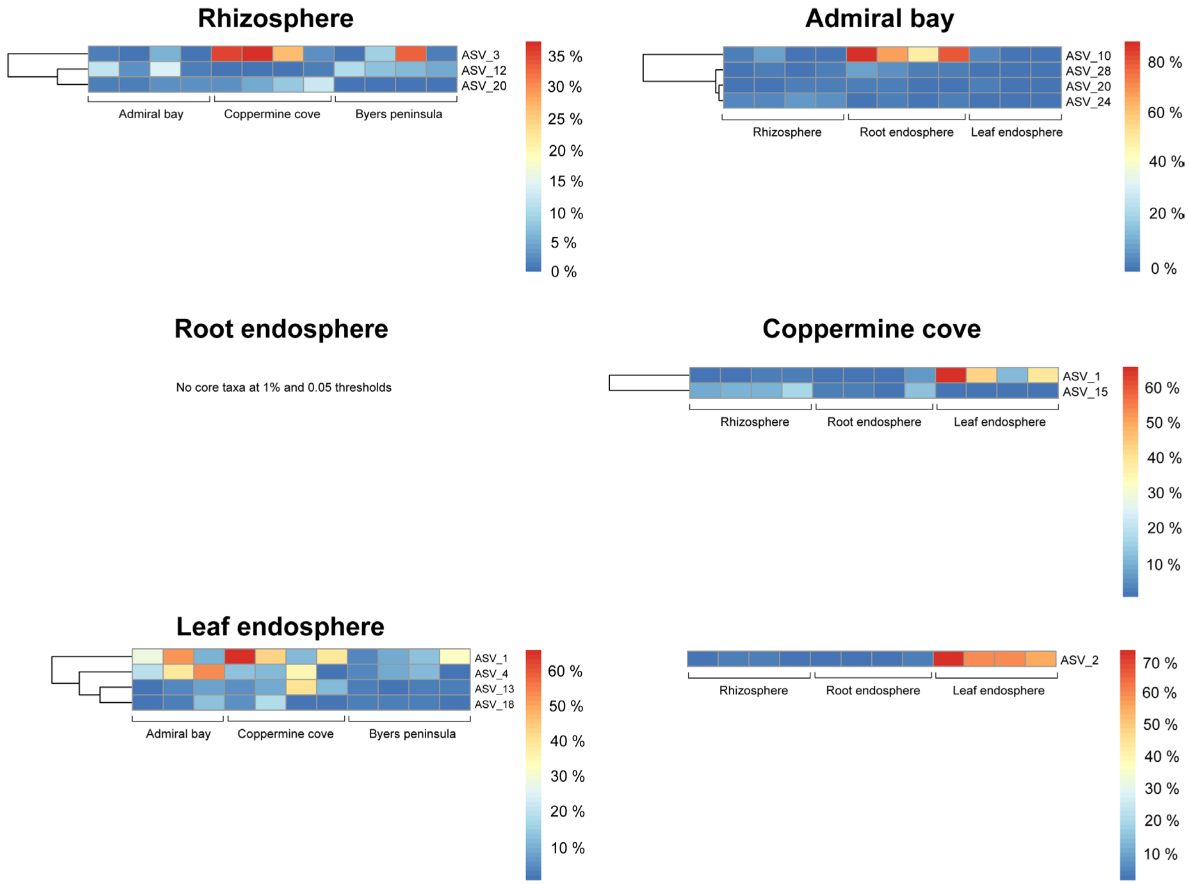

Supplementary Figure S31. Heatmap of relative abundances of fungal core taxa across plant compartments (rhizosphere, root endosphere, leaf endosphere) and sampling sites (Admiralty Bay, Coppermine Cove, Byers Peninsula). Only taxa meeting the ≥50% prevalence and ≥1% relative abundance thresholds are displayed. Color intensity represents relative abundance after CLR transformation.

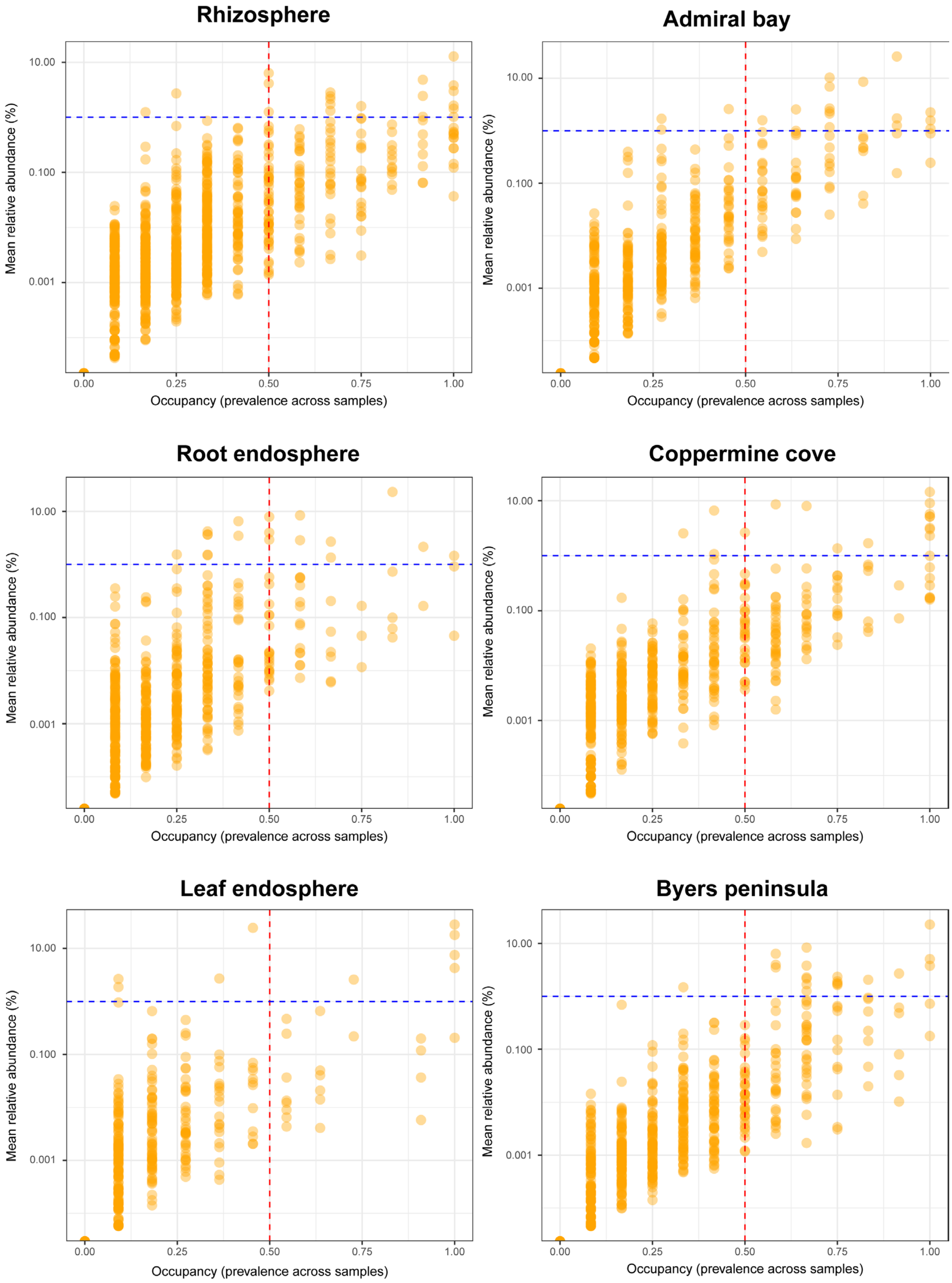

Supplementary Figure S32. Occupancy–abundance plot for fungal taxa across all samples. Each point represents an ITS zOTU colored by phylum-level classification. Core fungal taxa cluster in the high occupancy–high abundance region, reflecting their conserved presence across compartments and islands.

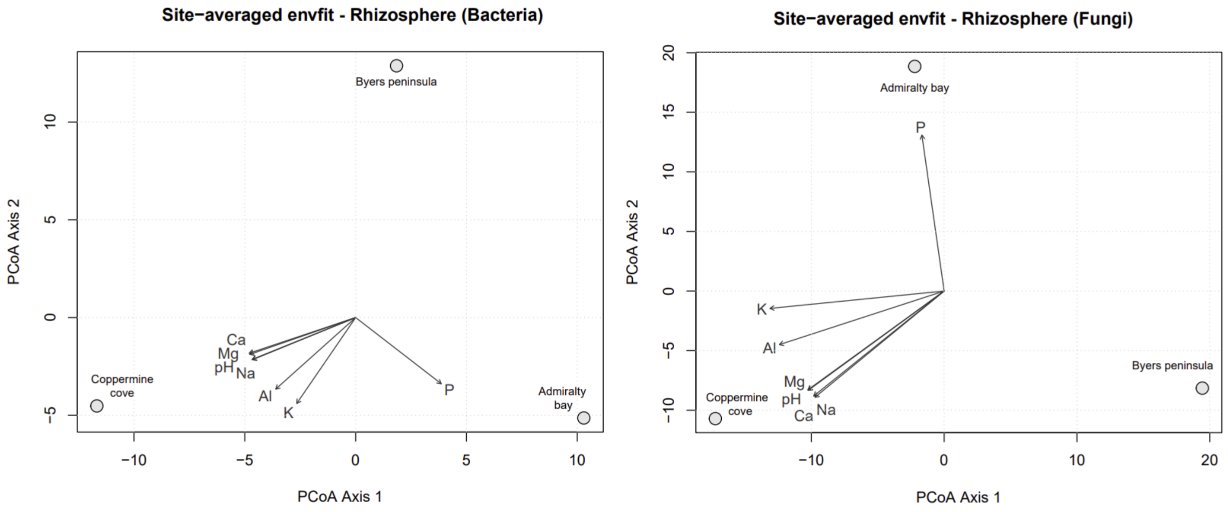

Supplementary Figure S33. Site-averaged *envfit* ordinations of bacterial (left) and fungal (right) rhizosphere communities of Deschampsia antarctica across the South Shetland Islands. Vectors represent soil variables (pH, Ca, Mg, Na, K, Al, P) fitted using *envfit* (vegan, R). Coppermine Cove aligns with base cations, Admiralty Bay with phosphorus enrichment, and Byers Peninsula shows weaker associations, indicating distinct edaphic gradients across sites.
